# Benzylphosphonic Acid‐Engineered Compact Self‐Assembled Monolayers for Bifacial Buried Interface Passivation in High‐Performance Inverted Perovskite Solar Cells

**DOI:** 10.1002/advs.202512117

**Published:** 2025-09-25

**Authors:** Liujiang Zhang, Meirong Fu, Xianyuan Jiang, Ziheng Zhang, Chenyue Wang, Zhenhuang Su, Bingchen He, Lin Tang, Guanhaojie Zheng, Xingyu Gao, Jianhua He

**Affiliations:** ^1^ The Institute for Advanced Studies Wuhan University Wuhan 430072 China; ^2^ Shanghai Synchrotron Radiation Facility Shanghai Advanced Research Institute Chinese Academy of Sciences Shanghai 201204 China; ^3^ Shanghai Institute of Applied Physics Chinese Academy of Sciences Shanghai 201800 China; ^4^ School of Physical Science and Technology ShanghaiTech University Shanghai 201210 China

**Keywords:** bifacial passivation, compact single SAM layer, Co‐SAM strategy, inverted perovskite solar cells

## Abstract

While [4‐(3,6‐Dimethyl‐9*H*‐carbazol‐9‐yl)butyl]phosphonic acid (Me‐4PACz) self‐assembled monolayers (SAMs) enable high‐performance inverted perovskite solar cells (PSCs), their sparse coverage on nickel oxide (NiOx) induces heterogeneous interfacial charge distribution at the buried perovskite interface. This increases non‐radiative recombination, ultimately limiting device performance. Herein, benzylphosphonic acid (BPPA) is added, a small molecule featuring a phosphonic acid group, into Me‐4PACz to construct a co‐assembled monolayer (Co‐SAM) with enhanced molecular ordering on NiOx. The resulting compact Co‐SAM hole transport layer (HTL) simultaneously improves electrical conductivity, hole mobility, and interfacial energy level alignment, facilitating efficient hole injection. Moreover, BPPA's phosphonic acid groups enable bifacial passivation: coordinating NiOx surface defects while chelating uncoordinated Pb^2+^ at the perovskite interface, significantly suppressing non‐radiative recombination. Optimized Me‐4PACz/BPPA‐based PSCs achieve a champion power conversion efficiency (PCE) of 26.35%, while retaining 90% of the initial efficiency after 3000 h in a nitrogen atmosphere without encapsulation. This molecular co‐assembly strategy concurrently refines HTL properties and buried interface passivation, providing a generalized approach for high‐efficiency, stable PSCs.

## Graphical abstract

Sparse Me‐4PACz SAM coverage on NiOx limits inverted PSC performance through interfacial recombination. Incorporating benzylphosphonic acid (BPPA) enables a compact Co‐SAM that enhances conductivity and hole mobility and improves energy alignment. BPPA's phosphonic acid groups bifacially passivate NiOx defects and Pb^2+^ ions at the buried interface. Co‐SAM PSCs achieve a champion 26.35% PCE with operational stability retention of 90% after 3000 h in a nitrogen atmosphere.

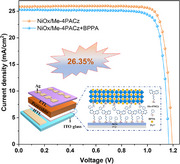

## 1 Introduction

Inverted perovskite solar cells have garnered significant research interest due to their simple structure and superior long‐term operational stability under illumination and thermal stress.^[^ ^1^, ^2^, ^3^ ^]^ Continuous advancements in hole transport layer (HTL) engineering and additive optimization strategies^[^ ^4^, ^5^, ^6^, ^7^ ^]^ have enabled these devices to surpass their conventional counterparts, achieving certified power conversion efficiencies (PCEs) of ≈27%.^[^ ^8^, ^9^ ^]^ However, the intrinsic instability of hole transport materials and the mismatch of interface energy levels remain fundamental bottlenecks restricting further enhancement of both efficiency and operational durability in inverted architectures.^[^ ^10^, ^11^, ^12^ ^]^ Addressing this dual challenge of extending operational longevity while maintaining high‐efficiency performance is crucial for their commercialization.^[^ ^13^ ^]^

In inverted perovskite solar cells, organic materials such as Poly [bis (4‐phenyl) (2,4,6‐trimethylphenyl)amine] (PTAA) are commonly used as the HTL.^[^ ^12^, ^14^ ^]^ However, their poor wettability, inadequate conductivity, and energy level mismatch with perovskites become the obstacles that hinder their wide usage for further improvement in cell efficiency.^[^ ^15^, ^16^ ^]^ Another class of HTL materials is inorganic oxides, such as nickel oxide (NiOx), which possesses excellent chemical stability and high optical transparency. However, these materials still suffer from problems such as high surface defect density, limited carrier extraction efficiency, and sensitivity to preparation processes, resulting in suboptimal PCE when used as HTL in inverted solar cells.^[^ ^17^, ^18^ ^]^ Several years ago, self‐assembled molecules (SAMs) emerged as efficient HTLs, which are ordered assemblies of organic compounds that include an anchoring group. This anchoring group forms a strong chemical bond with the hydroxyl groups on the surface of transparent conductive oxide electrodes.^[^ ^19^ ^]^ In addition, SAMs comprise an alkyl chain aligning perpendicularly to the substrate via van der Waals interactions, and a functional head group that tailors interfacial characteristics governing perovskite‐SAM interactions.^[^ ^20^, ^21^ ^]^ In recent years, SAMs containing phosphonic acid groups, such as [2‐(3,6‐Dimethoxy‐9*H*‐carbazol‐9‐yl)ethyl]phosphonic acid (MeO‐2PACz) and [4‐(3,6‐Dimethyl‐9*H*‐carbazol‐9‐yl)butyl]phosphonic acid (Me‐4PACz), have demonstrated the ability to form exceptional hole‐selective interfaces in inverted perovskite solar cells, achieving remarkable efficiencies.^[^ ^21^, ^22^, ^23^, ^24^, ^25^ ^]^

While NiOx and SAMs are commonly used as HTLs in inverted perovskite solar cells, critical challenges–including unfavorable energy level alignment, inefficient charge extraction, numerous interfacial defects, and limited chemical stability–remain to be overcome.^[^ ^26^, ^27^ ^]^ For instance, the steric hindrance of the carbazole core in the SAMs limits the formation of SAMs on the substrate. Furthermore, the low solubility of SAMs and their insufficient chemical bonding with NiOx result in gaps on the substrate. These weaknesses, combined with the imperfect deposition conditions and substrate morphology, lead to low surface coverage on indium tin oxide (ITO). Incomplete or uneven SAM coverage on the substrate facilitates undesired direct contact between the active perovskite layer and the electrode. This results in severe non‐radiative charge recombination, which threatens the operational stability of the device and jeopardizes its application in perovskite photovoltaic cells.^[^ ^28^, ^29^ ^]^ Therefore, a uniformly distributed and well‐covered SAM layer is crucial for enhancing interface stability and device performance.^[^ ^27^, ^30^, ^31^, ^32^ ^]^ Currently, several strategies have been reported to address issues such as the insufficient coverage of Me‐4PACz on the substrate, its tendency to aggregate, and its poor wettability with the perovskite precursor.^[^ ^8^, ^33^, ^34^ ^]^

**Figure 1:**
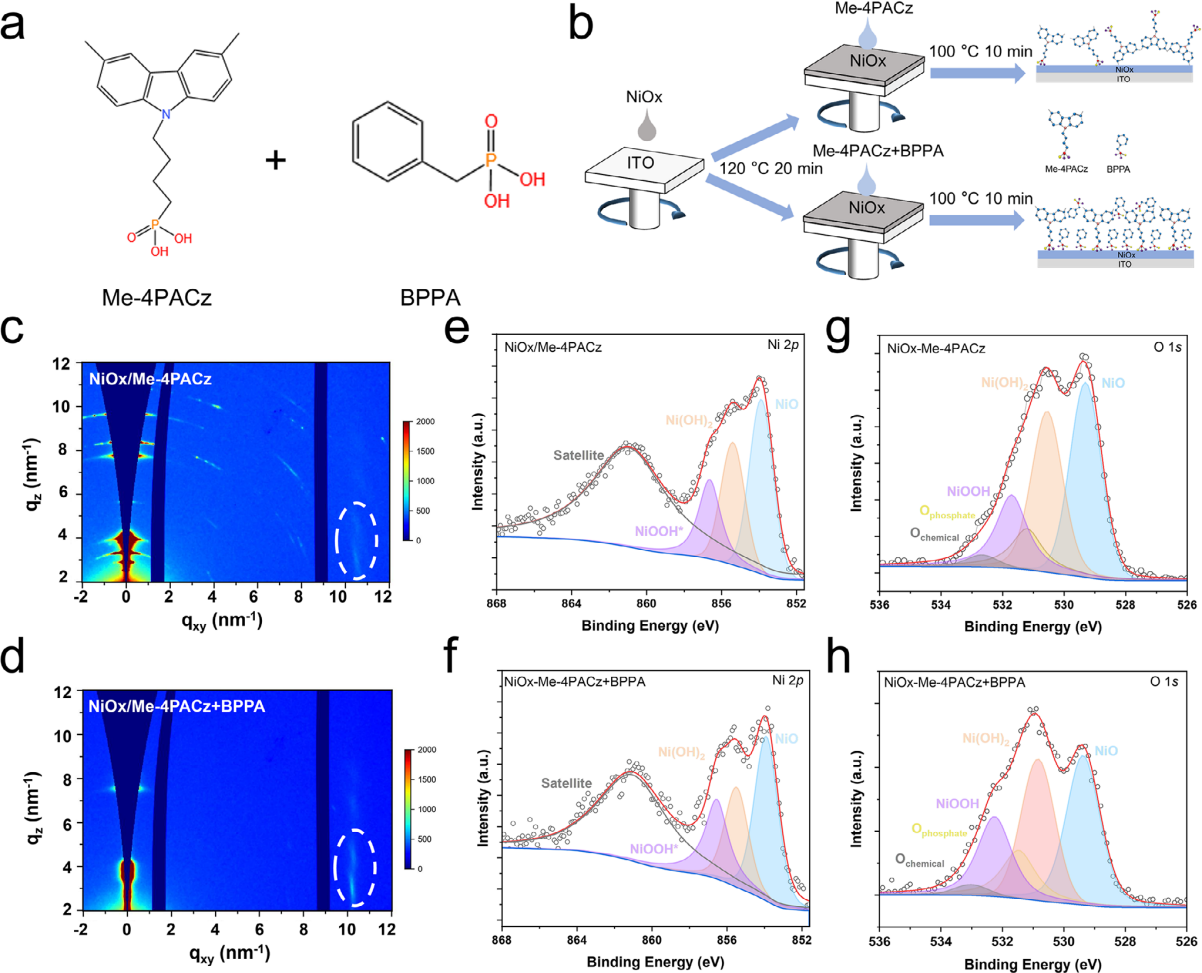
Influences of BPPA on HTL. a) Molecular structures of Me‐4PACz and BPPA. b) Schematic diagram of the deposition steps of SAM (Me‐4PACz) and Co‐SAM on the NiOx substrate. 2D GIWAXS patterns of c) Me‐4PACz and d) Me‐4PACz+BPPA deposited on Si/NiOx substrates. The Ni 2*p* XPS spectra for e) NiOx/Me‐4PACz and f) NiOx/Me‐4PACz+BPPA. The O 1*s* XPS spectra for g) NiOx/Me‐4PACz and h) NiOx/Me‐4PACz+BPPA.

In this work, we fabricated perovskite solar cells with passivated buried interface defects using a co‐assembled monolayer (Co‐SAM), where benzylphosphonic acid (BPPA) and Me‐4PACz co‐assemble to form a uniform and densely packed monolayer on the substrate (**Figure** **1**a). On the one hand, the addition of BPPA enables Me‐4PACz to adopt a more ordered distribution with improved vertical preferential orientation on the NiOx substrate. Critically, interactions between the phosphonic acid groups in BPPA and NiOx increase the proportion of Ni^3+^, suppress NiOx surface defects, and enhance the conductivity and hole mobility of the HTL. Furthermore, the Co‐SAM enhances both the coverage and uniformity of SAMs on NiOx, thereby enhancing HTL smoothness and wettability toward the perovskite precursor solution. Additionally, BPPA incorporation optimizes energy level alignment between the HTL and perovskite film, preventing carrier accumulation at the interface while facilitating carrier transport. On the other hand, the phosphonic acid groups passivate interfacial uncoordinated Pb^2^⁺ with reduced lead iodide content, thereby suppressing non‐radiative recombination. These improvements collectively create favorable conditions for the growth of high‐quality perovskite films. Thus, the Co‐SAM modified HTL achieves efficient charge transport with bifacial passivation functionality. Consequently, the Co‐SAM‐treated device achieved a remarkable champion power conversion efficiency of 26.35%, and the unencapsulated device retained 90% of its initial efficiency after 3000 h of storage in a nitrogen (N_2_)‐glovebox.

## 2 Results and Discussion

### 2.1 Hole Transport Layer

We prepared a Co‐SAM by spin‐coating a blend of BPPA and Me‐4PACz (3:5 w/w) onto the NiOx substrates. While Me‐4PACz alone fails to form dense monolayers on NiOx due to its steric hindrance, the addition of BPPA promotes uniform Co‐SAM formation with near‐complete substrate coverage, as illustrated in Figure 1b. To elucidate the mechanism, the Gaussian‐calculated electrostatic potential map (Figure S1, Supporting Information) reveals that BPPA's phosphonic acid group exhibits stronger electron‐donating capacity than Me‐4PACz, with its negatively charged character (orange regions) facilitating enhanced charge donation to the NiOx surface.

To investigate the micro‐structural properties of the SAMs deposited on NiOx, synchrotron‐based grazing incidence wide‐angle X‐ray scattering (GIWAXS) was employed to compare the crystallinity and molecular orientation of NiOx/Me‐4PACz and NiOx/Me‐4PACz+BPPA films. As shown in **Figure** **1**c,d, the GIWAXS results reveal that Me‐4PACz exhibits a face‐on preferential orientation, with multiple Debye‐Scherrer rings appearing in a relatively disordered distribution.^[^ ^28^, ^35^ ^]^ However, when BPPA is mixed into Me‐4PACz, most Debye‐Scherrer rings disappear, particularly in the out‐of‐plane direction. The intensity of the diffraction streak ≈q = 7.5 nm^−1^ is noticeably weakened. More importantly, the in‐plane diffraction intensity of the Co‐SAM film increases ≈q = 10 nm^−1^ (highlighted by the dashed white circle). The calculated interplanar spacing of ≈0.6 nm from the diffraction peak ≈q = 11 nm^−1^ matches the lateral packing distance of Me‐4PACz molecules on the substrate, indicating that BPPA treatment promotes a more ordered molecular packing structure.^[^ ^35^, ^36^ ^]^

To further quantify the analysis, we extracted 1D GIWAXS profiles along both the out‐of‐plane and in‐plane directions from 2D GIWAXS patterns of the two samples, as shown in Figure S2 (Supporting Information). From the 1D GIWAXS spectra, it is evident that the out‐of‐plane diffraction peaks not only become fewer but also weaker. In contrast, the in‐plane diffraction peaks are significantly enhanced. These results indicate that adding BPPA to Me‐4PACz effectively suppresses Me‐4PACz aggregation on NiOx, facilitating the formation of a more ordered Co‐SAM. Under the influence of the smaller BPPA molecules, Me‐4PACz anchors more uniformly onto NiOx.^[^ ^35^, ^37^ ^]^ By filling the gaps between Me‐4PACz molecules, the smaller phosphonic acid molecules optimize molecular interactions within the SAM layer. This leads to a more ordered distribution of Me‐4PACz on the NiOx surface, enabling the formation of a well‐packed, full‐coverage monolayer anchored via phosphonic acid groups.^[^ ^31^ ^]^

In the following, we utilized X‐ray photoelectron spectroscopy (XPS) to investigate the chemical species of Co‐SAM on NiOx. Careful fitting of the XPS spectra was carried out, and the peak positions and ratios of all elements obtained from the fittings are summarized in Tables S1 and S2 (Supporting Information). Typically, Ni^3+^ is advantageous for hole transport and mainly originates from Ni vacancies at the grain boundaries of NiOx.^[^ ^7^, ^38^ ^]^ By deconvoluting the Ni 2*p* peaks (Figure 1e,f), we categorized the Ni 2*p* spectrum into NiO, Ni(OH)_2_, and NiOOH.^[^ ^39^ ^]^ Comparing Co‐SAM with Me‐4PACz on NiOx, the proportion of Ni^3+^ increases from 24.6% to 28.5%, that of Ni^2+^ decreases from 30.7% to 27.4%, while that of NiO remained largely unchanged. Simultaneously analyzing the O 1*s* spectrum (Figure 1g,h) categorized it into NiO, Ni(OH)_2_, and NiOOH, oxygen in phosphonate groups, and chemically bound oxygen.^[^ ^39^ ^]^ We observed that the proportion of NiOOH increases significantly in Co‐SAM on NiOx, while that of Ni(OH)_2_ slightly decreases. Additionally, the proportion of oxygen corresponding to phosphonic acid also increases in Co‐SAM on NiOx, which is consistent with the increased proportion of phosphorus in the P 2*p* spectrum (Figure S3 and Table S3, Supporting Information). This suggests that adding BPPA enhances the interactions between Me‐4PACz and the NiOx substrate, facilitating conversion of Ni(OH)_2_ to NiOOH. In NiOx films, Ni^2+^ is typically the predominant oxidation state. However, the strong electronegativity of phosphonic acid groups allows their oxygen atoms to coordinate with Ni^2+^, facilitating electron withdrawal and promoting the oxidation of Ni^2+^ to Ni^3+^. This oxidation process increases Ni^3+^ concentration, thereby enhancing the p‐type conductivity of NiOx.^[^ ^40^ ^]^ This indicates that more phosphonic acid is adsorbed on the NiOx surface, resulting in a more uniform distribution of phosphonic acid on NiOx. Generally, a higher Ni^3+^ concentration contributes to improved conductivity and enhanced hole transport capability of the HTL.^[^ ^38^ ^]^

**Figure 2:**
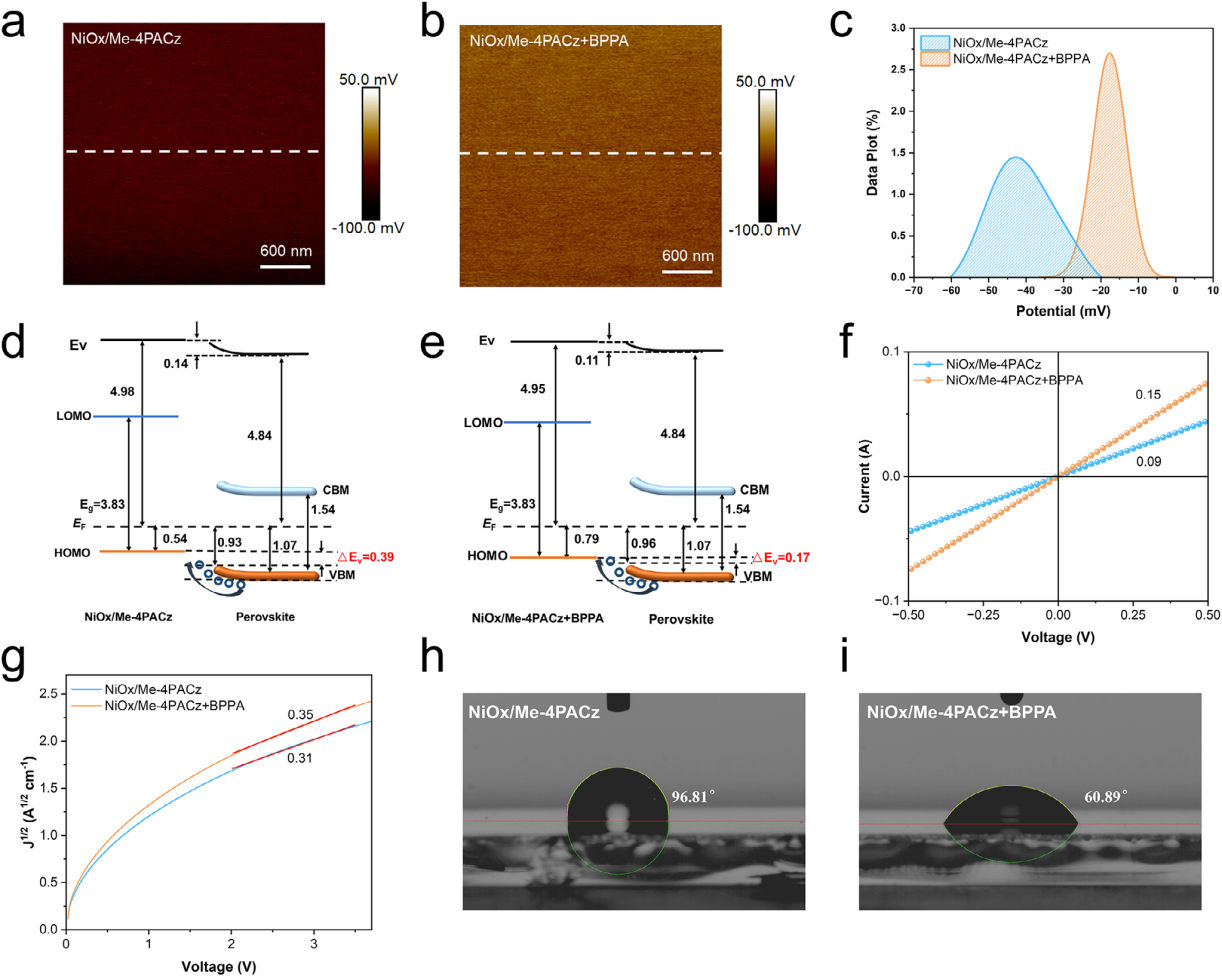
Influences of BPPA on surface morphology and electrical properties of HTL. KPFM images of a) NiOx/Me‐4PACz and b) NiOx/Me‐4PACz+BPPA. c) CPD distributions of NiOx/Me‐4PACz and NiOx/Me‐4PACz+BPPA. Schematic diagram of energy level alignment for d) NiOx/Me‐4PACz and e)NiOx/Me‐4PACz+BPPA with perovskite. The conductivity f) and the hole mobility g) of NiOx/Me‐4PACz and NiOx/Me‐4PACz+BPPA. Contact angles of the perovskite precursor solution on h) NiOx/Me‐4PACz and i) NiOx/Me‐4PACz+BPPA.

Next, we evaluated the roughness and electrical properties of Co‐SAM on the NiOx surface. Atomic force microscopy (AFM) revealed that the surface roughness of Me‐4PACz+BPPA on NiOx (5.73 nm) became smaller than that of Me‐4PACz alone on NiOx (6.13 nm) (Figure S4a,b, Supporting Information). Additionally, the height variation curve along a specific line on the film surface is smoother (Figure S4c, Supporting Information). The surface roughness distribution (Figure S4d, Supporting Information) demonstrates that NiOx/Me‐4PACz+BPPA yields a more uniform and smoother Co‐SAM interface, as evidenced by more narrowly distributed roughness values. This improved interfacial morphology is expected to suppress structural inhomogeneities and reduce defect density within the HTL. At the same time, we used Kelvin probe force microscopy (KPFM) to measure the surface potential difference of the NiOx/Me‐4PACz film and the NiOx/Me‐4PACz+BPPA film (**Figure** **2**a,b). The Co‐SAM exhibits a higher surface potential with a more uniform distribution. Potential distribution diagrams (Figure 2c) confirm a narrower contact potential difference (CPD) distribution for NiOx/Me‐4PACz+BPPA, indicating enhanced potential uniformity. These results demonstrate a smoother film surface and a densely packed SAM layer.^[^ ^41^ ^]^ Furthermore, to corroborate the enhanced surface coverage of the Co‐SAM on the NiOx substrate, XPS analysis was performed on both NiOx/Me‐4PACz and NiOx/Me‐4PACz+BPPA films (Tables S1–S3, Supporting Information). The atomic ratio of P 2*p* to Ni 2*p* was quantified to further support this observation (Note S1, Supporting Information). The resulting ratio for NiOx/Me‐4PACz+BPPA (1.013) was significantly greater than that of the NiOx/Me‐4PACz (0.381), corresponding to a 166% increase. These findings clearly demonstrate that the incorporation of BPPA effectively improves the packing density of Me‐4PACz on NiOx, leading to the formation of a more continuous and compact SAM monolayer.^[^ ^42^ ^]^

We used ultraviolet photoelectron spectroscopy (UPS) to measure the valence band maximum (VBM) and work function (WF) of the HTL and perovskite films (Figure S5, Supporting Information). The VBM and E_F_ are 0.57 and 4.98 eV for NiOx/Me‐4PACz, respectively, and 0.79 and 4.95 eV for NiOx/Me‐4PACz+BPPA, respectively. Meanwhile, the perovskite film exhibits a VBM of 1.07 eV with an E_F_ of 4.84 eV. The bandgaps of 3.83 eV (HTL) and 1.54 eV (perovskite) can be obtained from ultraviolet visible (UV–vis) measurements (Figure S6, Supporting Information). Based on these data, we constructed the HTL/perovskite energy level alignment diagram (Figure 2d,). The HOMO onset of the HTL lies above the perovskite VBM for both SAM and Co‐SAM, facilitating hole transfer. The hole injection barrier (ΔEv)–defined as the energy difference between the perovskite VBM and HTL HOMO onset–promotes hole injection, but excessive ΔEv causes interfacial energy loss and reduces open‐circuit voltage (V_OC_).^[^ ^43^ ^]^ We observe ΔEv values of 0.17 eV for NiOx/Me‐4PACz+BPPA/perovskite versus 0.39 eV for NiOx/Me‐4PACz/perovskite. The smaller ΔEv indicates superior energy level alignment between NiOx/Me‐4PACz+BPPA and perovskite, promoting efficient hole transfer from perovskite to HTL. This mitigates interfacial carrier accumulation and enhances device V_OC_. Furthermore, UV–vis absorption spectroscopy was performed on NiOx/Me‐4PACz and NiOx/Me‐4PACz+BPPA (Figure S7, Supporting Information), revealing that the incorporation of BPPA does not alter the optical absorption characteristics of the HTL.

To investigate the effect of BPPA addition to the SAM on charge transfer in the NiOx/Me‐4PACz layer, we measured conductivity and hole mobility using ITO/NiOx/Me‐4PACz (with/without BPPA)/Ag devices. Conductivity (*σ*) was calculated as: (1) σ = d / AR, where d, A, and R represent the HTL thickness, the film area, and the resistance, respectively.^[^ ^44^ ^]^ It is shown from Figure 2f that the conductivity increases significantly from 2.48 ×10^−3^ mS cm^−1^ (NiOx/Me‐4PACz) to 4.13 × 10^−3^ mS cm^−1^ (NiOx/Me‐4PACz+BPPA). Hole mobility was derived from current density‐voltage curves (Figure 2g) fitted to the Mott–Gurney law: (2) J = (9/8) ε_0_ε_r_µ V^2^/L^3^, where J, ε_0_, ε_r_, L, µ represent the current density, the vacuum permittivity, the permittivity of NiOx/Me‐4PACz, the film thickness, and the mobility, respectively.^[^ ^45^ ^]^ The hole mobility increases from 5.94 × 10^−7^ cm^2^ V^−1^ s^−1^ (NiOx/Me‐4PACz) to 7.57 × 10^−7^ cm^2^ V^−1^ s^−1^ (NiOx/Me‐4PACz+BPPA). Thus, incorporating BPPA into Me‐4PACz to form a Co‐SAM enhances both HTL conductivity and hole mobility. Contact angle measurements (Figure 2h,i) show the perovskite precursor contact angle decreases from 96.81° (NiOx/Me‐4PACz) to 60.89° (NiOx/Me‐4PACz+BPPA), indicating enhanced wettability. This enhancement arises from the presence of additional polar phosphonic acid groups derived from BPPA at the surface, which promotes better perovskite precursor spreading.^[^ ^46^, ^47^ ^]^ Improved wettability enhances perovskite film coverage and morphological quality on the HTL. Combined with the more uniform HTL surface and improved energy level alignment, this facilitates effective hole transfer at the HTL/perovskite interface while reducing charge accumulation.

In summary, the uniform and densely packed Co‐SAM presents improved vertical stacking order and uniform distribution of Me‐4PACz, enhancing hole mobility while reducing charge carrier recombination at the SAM/perovskite interface. The strong electron‐withdrawing capability of the phosphonic acid groups promotes the oxidation of Ni^2+^ to Ni^3+^, thereby enhancing the overall conductivity and hole mobility of the HTL. Moreover, the incorporation of the more polar BPPA yields both a more homogeneous surface potential distribution across the HTL and improved wettability for the perovskite precursor solution. In addition, improved energy level alignment will promote effective hole transfer at the HTL/perovskite interface while reducing interface charge accumulation.^[^ ^48^, ^49^, ^50^ ^]^

### 2.2 Perovskite Films

**Figure 3:**
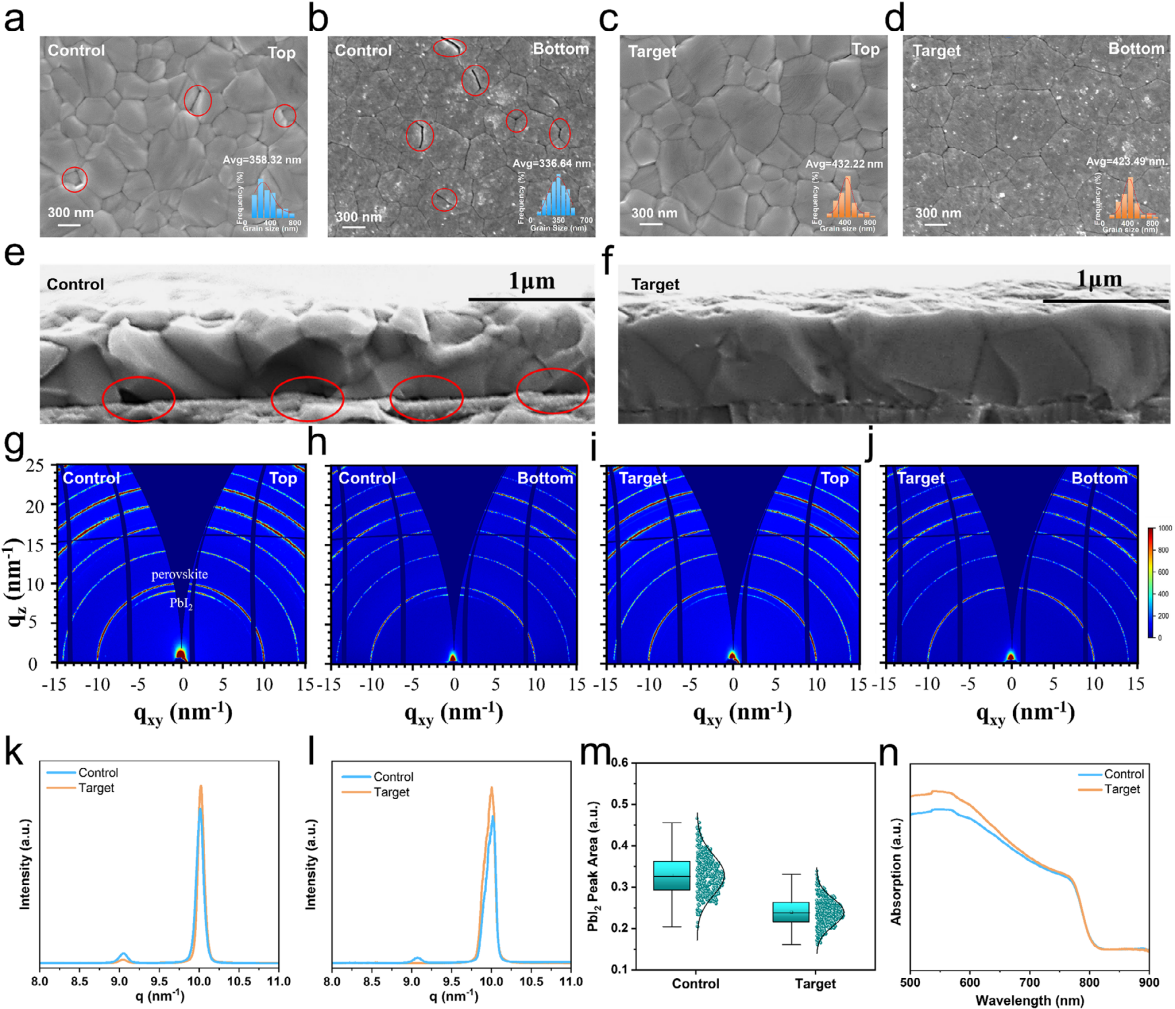
Influences of BPPA on film morphology and microstructure of perovskite films. The SEM images of the surface a, c), of the buried interface b,d), and cross‐sectional SEM images e,f) of the control and target perovskite films. 2D GIWAXS patterns at the surface g, i) and the buried interface h, j) of the control and target perovskite films. k) The top and l) bottom radial integration plot of 2D GIWAXS patterns of the two films. m) Statistical distribution chart of the PbI_2_ peak area for the control and target films. n)The UV–vis absorption spectra of the control and target films.

To study the influence of Co‐SAM on the perovskite films formed by adding BPPA into Me‐4PACz, we investigated their morphology, crystal structure, and optical properties. In the following, perovskite films grown on NiOx/Me‐4PACz and on NiOx/Me‐4PACz+BPPA are termed control and target, respectively. We first examined the perovskite surface and buried interface morphology using scanning electron microscopy (SEM). To access the buried interface, we coated the perovskite films with UV‐curable glue, then peeled them off to expose the buried interface of the perovskite films (Figure S8, Supporting Information).^[^ ^51^ ^]^ **Figure** **3**a–d shows the SEM images of the surface and buried interface of perovskite films grown on different substrates. It is shown that the target film surface shows a larger average grain size (432.22 vs control: 358.32 nm), with fewer cracks per hole. At the buried interface, target grains (average of 423.49 nm) are notably larger than control grains (average of 336.64 nm). Due to hydrophobicity and poor wettability, the control's buried interface exhibits nanogaps and cracks at grain boundaries, while the target interface is compact and smooth. The smoother contact between the perovskite film and the HTL, combined with the vertical grain orientation, enhances carrier transport and reduces non‐radiative recombination losses at the interface. Additionally, PbI_2_ appears more dispersed and sparser at the target interface, contrasting with significant PbI_2_ aggregation in the control, suggesting a higher PbI_2_ concentration there. The primary cause for the reduced amount of PbI_2_ observed at the buried interface in the SEM images may be attributed to the passivation effect of BPPA, which mitigates the uncoordinated PbI_2_ at the buried interface of the film. Cross‐sectional SEM (Figure 3e,f) reveals more intimate contact between the target film and HTL, while the control shows pores and uneven contact. The target film also features vertically aligned grains. AFM also shows that the target film surface is smoother with a roughness of 16.2 nm than the control film with that of 24.8 nm (Figure S9, Supporting Information), which facilitates improved contact between the perovskite film and the electron transport layer (ETL). All these pieces of evidence indicate that the presence of Co‐SAM promotes grain growth of the perovskite films, resulting in a smoother film with larger, denser grains and fewer defects.

The improved perovskite film morphology induced by the Co‐SAM should be related to enhanced crystallinity. Indeed, XRD spectra show that all the perovskite diffraction peaks are enhanced slightly with the perovskite crystal structure unaltered by Co‐SAM (Figure S10, Supporting Information). To further probe crystallinity, we employed GIWAXS at both surface and buried interfaces.^[^ ^52^ ^]^ Figure 3g–j displays the 2D GIWAXS patterns of the perovskite films’ surface and buried interface at an incidence angle of 0.3°, all exhibiting similar Debye–Scherrer rings characteristic of highly crystalline perovskite films. The ring ≈q = 10.0 nm^−1^ corresponds to the perovskite (001) plane, while that ≈q = 9.0 nm^−1^ corresponds to the PbI_2_ (001) plane. It is clear that the (001) diffraction ring of the target perovskite film at both the surface and the buried interface is brighter than its counterpart of the control film; however, no distinct preferential orientation is observed in all the diffraction patterns. Notably, the PbI_2_ diffraction ring weakens significantly for the target film both at the surface and the buried interfaces. We further integrated the Debye–Scherrer rings ≈q = 9.0 and 10.0 nm^−1^ from the 2D patterns to obtain 1D profiles, which are reported in Figure 3k for those at the surface and in Figure 3l for those at the buried interface. Both surface and buried interfaces of the target film show a slightly higher (001) perovskite peak and lower PbI_2_ peak versus the control film. This confirms that the Co‐SAM enhances perovskite crystallinity at both the surface and the buried interface while BPPA addition efficiently passivates the uncoordinated PbI_2_, reducing overall PbI_2_ content in the films. These conclusions align with SEM observations of improved film quality.

To evaluate the in‐plane uniformity of perovskite crystallization and PbI_2_ content at the buried interface, we measured *µ*‐GIWAXS scanning^[^ ^53^ ^]^ of perovskite films at 3° incidence. Figure S11 (Supporting Information) presents 𝜇‐GIWAXS mapping of the perovskite (001) peak area within a 5 × 5 mm^2^ area for target and control films with derived statistics. Figure S12 (Supporting Information) presents PbI_2_ (001) peak area 𝜇‐GIWAXS mapping on both films, with statistics in Figure 3m. Both perovskite and PbI_2_ distributions are more uniform on the target film, showing narrower statistical spreads. Notably, PbI_2_ content is reduced in the target film. Quantitative mean and standard deviation (SD) values for the two peak areas are summarized in Table S4 (Supporting Information). The control film shows perovskite (001) mean peak area = 9.76 (SD: 1.42) and that for PbI_2_ = 0.33 (SD: 0.05). In contrast, the target film exhibits perovskite (001) mean peak area = 9.87 (SD: 1.23) and that for PbI_2_ = 0.24 (SD: 0.03). These results confirm improved perovskite crystallization with reduced PbI_2_ at the buried interface. Lower SD values for both components evidence enhanced uniformity in the target film. Thus, BPPA addition to Me‐4PACz enhances buried interface crystallization quality while reducing uncoordinated PbI_2_ content.^[^ ^54^ ^]^

To understand the improved film quality on the Co‐SAM, in situ GIWAXS was carried out during annealing of perovskite films on NiOx/Me‐4PACz and NiOx/Me‐4PACz+BPPA (Figure S13a,b, Supporting Information). Both films contained δ‐phase and α‐phase perovskite before annealing. During annealing, a similar δ‐to‐α phase transition occurred on both substrates. To be more quantitative, Figure S13c (Supporting Information) plots α‐phase and δ‐phase peak areas versus annealing time. Notably, the target film initially showed more δ‐phase content but completed the transition to α‐phase ≈10 s earlier than the control film. The target film also exhibited more initial *α*‐phase content. These observations explain enhanced perovskite crystallization in annealed target films, attributable to improved wettability accelerating the α‐phase transition.^[^ ^8^, ^55^ ^]^ UV–vis absorption spectra show identical absorption edges for both films, but the target film exhibits notably enhanced absorption (Figure 3n). This confirms unchanged perovskite bandgap but improved crystallinity with larger grains, consistent with SEM and GIWAXS results.

**Figure 4:**
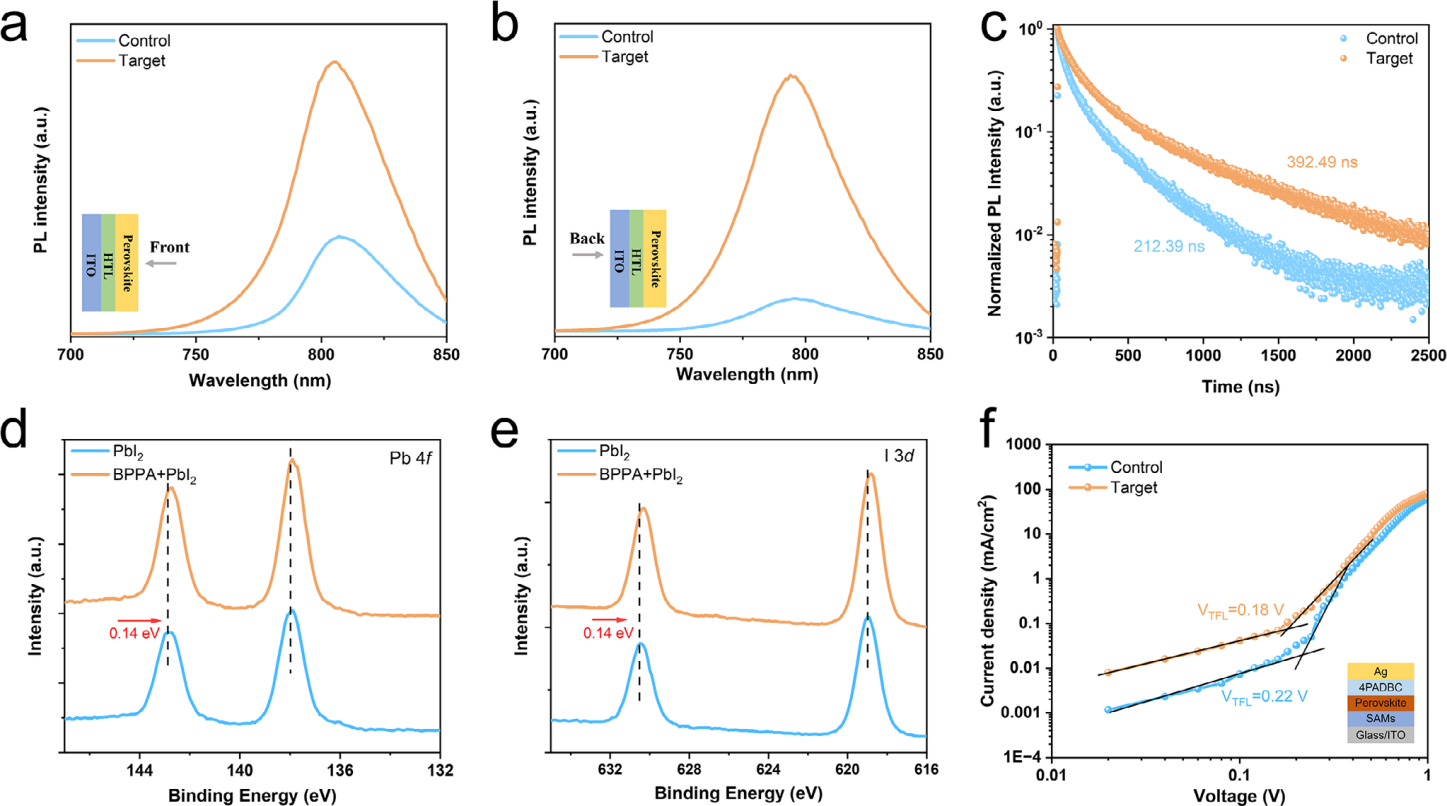
Investigation of carrier transport and defect states, as well as the interactions between BPPA and lead iodide. The PL spectra from the a) front side and from the b) back side of control and target films. c) The TRPL spectra of control and target films. XPS spectra of d) Pb 4*f* and e) I 3*d* for PbI_2_ films with/without BPPA, respectively. f) SCLC of the hole‐only devices.

To investigate Co‐SAM's effect on the carrier transport of perovskite films, we measured photoluminescence (PL) and time‐resolved PL (TRPL) of perovskite films on different ITO/HTL substrates. Front‐side PL (light incident from film surface) and back‐side PL (light incident from ITO substrate) spectra were collected. As shown in **Figure** **4**a, front‐side PL reveals a significantly higher peak for the target film versus the control, indicating superior film quality and lower defect density. Figure 4b shows back‐side PL spectra where this peak height difference further increases, demonstrating effective passivation of perovskite at the buried interface with suppressed non‐radiative recombination. TRPL spectra (Figure 4c) were fitted with a biexponential decay model (parameters in Table S5, Supporting Information).^[^ ^56^ ^]^ The average carrier lifetime increases by ≈85% from 212.39 to 392.49 ns with BPPA addition. This confirms Co‐SAM effectively passivates buried interface defects and suppresses non‐radiative recombination.^[^ ^56^ ^]^ We attribute this improvement to the uniform coverage of the high‐quality Co‐SAM on NiOx.

To understand the improved perovskite film quality at the buried interface on Co‐SAM, XPS measurements were performed to look for possible interactions between BPPA and lead iodide. For such measurements, BPPA was added into a lead iodide solution, which was spin‐coated to form a thin film. The Pb 4*f* and I 3*d* XPS spectra of the PbI_2_ films with/without BPPA are reported in Figure 4d,e, respectively. Compared to PbI_2_ films, the BPPA addition shifts both Pb 4*f* and I 3*d* to lower binding energies. Quantitatively, the Pb 4*f_5/2_* peak at 142.87 eV and the Pb 4*f*_7/2_ peak at 137.97 eV shift to 142.73 and 137.83 eV, respectively. Similarly, the I 3*d*_3/2_ peak at 630.49 eV and the I 3*d*_5/2_ peak at 618.97 eV shift to 630.35 and 618.83 eV, respectively. In addition, we performed XPS measurements on the buried interfaces (mechanical exfoliation as shown in Figure S8, Supporting Information) of perovskite films deposited on NiOx/Me‐4PACz and NiOx/Me‐4PACz+BPPA substrates to examine the influence of BPPA incorporation on the binding energies of Pb and I. As shown in Figure S14 (Supporting Information), shifts of the Pb 4*f* and I 3*d* peaks toward lower binding energies are evident, consistent with the trend identified in BPPA‐doped PbI_2_ films. These results confirm the presence of interactions between BPPA and both Pb^2+^ and I^−^ ions.

The Co‐SAM improves the quality of the perovskite film, which should lead to reduced defect states. To analyze the trap state density of perovskite thin films on different substrates, pure hole devices with a structure of ITO/NiOx/Me‐4PACz or Co‐SAM/perovskite/4PADBC/Ag were fabricated to measure space charge limited current (SCLC) as shown in Figure 4f. The trap state density is calculated using the following formula:^[^ ^57^ ^]^ (3) n_trap_  =  2εε_0_V_TFL_/(eL^2^), where ε_0_ is the vacuum permittivity, ε is the dielectric constant of the perovskite, e is the elementary charge, and L is the thickness of the perovskite thin film. From the measurements, the target device exhibits a trap‐filled limit voltage V_TFL_ of 0.18 V with a trap state density of 1.04 × 10^15^ cm^−3^, lower than a V_TFL_ of 0.22 V with a trap state density of 1.27 × 10^15^ cm^−3^ for the control device. These observations confirm that Co‐SAM optimizes the buried interface with film quality enhanced and the defect density lowered in perovskite films.

### 2.3 Perovskite Solar Cells

**Figure 5:**
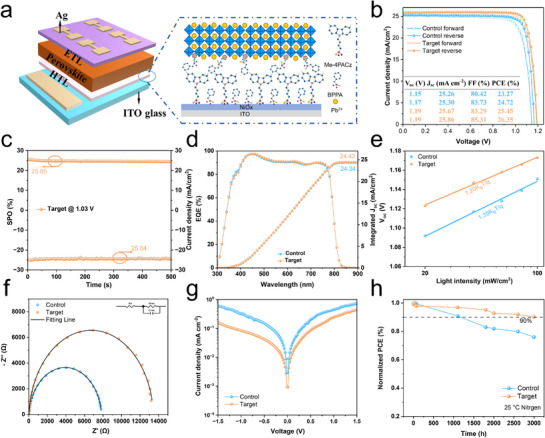
Enhanced PSCs' performance by Co‐SAM. a) Schematic of the present inverted PSC structure, and the left side highlights the HTL structure on the ITO substrate. b) The J–V curves of the forward and reverse direction scans of the champion target and control PSCs. c) Steady‐state efficiency and stabilized photocurrent for PSCs on Co‐SAM at a maximum power output point at 1.03 V. d) EQE spectra and integrated current densities for the champion target and control PSCs. e) Light intensity dependence of V_OC_ of control and target PSCs. f) The Nyquist plots of control and target PSCs. g) Dark J–V curves of control and target PSCs. h) The normalized PCE evolution of unencapsulated control and target PSCs stored in nitrogen.

In the following, we will demonstrate the improved performance of perovskite solar cells based on Co‐SAM. Typical inverted PSCs with a structure of ITO/NiOx/Me‐4PACz (with or without BPPA)/perovskite/C_60_/BCP/Ag were fabricated as illustrated in **Figure** **5**a, where on the left side a schematic highlights the HTL structure on the ITO substrate. To verify the optimal mixing ratio of BPPA and Me‐4PACz (3:5 w/w), Figure S15 (Supporting Information) reports the statistical distribution of PSCs performance based on Me‐4PACz mixed with different amounts of BPPA. It is clear that a BPPA:Me‐4PACz ratio of 3:5 w/w yields the best average device performance, which is also evidenced in Figure S16 (Supporting Information) reporting the optimal efficiency curves and photovoltaic parameters for the devices at various BPPA mixing concentrations. To investigate the hysteresis effects, the forward and reverse scanning current density–voltage (J–V) curves of the control and target champion PSCs are present in Figure 5b. In comparison with the control PSC in the reverse scan, the target PSC’ PCE increases from 24.72% to 26.35%, with the V_OC_ rising from 1.17 to 1.19 V, short‐circuit current density (J_SC_) from 25.30 to 25.86 mA cm^−2^, and the fill factor (FF) from 83.73% to 85.31%. Similarly, in the forward scan, the target PSC exhibited a boosted PCE of 25.45%, up from 23.27% for the control PSC. It is also clear that the hysteresis factor^[^ ^27^ ^]^ (HF, Note S2, Supporting Information) decreases significantly from 5.9% to 3.4%, attributed to improved carrier transport and reduced recombination. To further prove the enhanced PSC performance, Figure 5c reports a steady‐state output efficiency of 25.85% with a stable J_SC_ of 25.04 mA cm^−2^ for the target PSC, which outperforms the control PSC shown in Figure S17 (Supporting Information). The EQE spectra in Figure 5d also show that the integrated current of the target PSC increases to 24.42 mA cm^−2^ from 24.34 mA cm^−2^ for the control PSC, in line with J–V measurements. It is also noticed that the analysis of the EQE spectra yields a bandgap of 1.54 eV, which is consistent with the result obtained from UV–vis measurements (Figures S18,S6, Supporting Information). To check the PSC performance reproducibility, Figure S19a–d (Supporting Information) reports the statistical distribution of PCE, V_OC_, Jsc, and FF for all fabricated control and target PSCs, respectively. While PCE is enhanced notably, the improvement in V_OC_ is significant, attributed to better alignment of the interface energy levels. It is also noticed that FF is also enhanced notably. More remarkably, the narrowing statistical distributions of the J–V parameters in Figure S19 (Supporting Information) demonstrate improved PSCs reproducibility.

To elucidate the dominant charge recombination pathways in different devices, we measured the light intensity dependence of V_OC_ for a control PSC and a target PSC (Figure 5e). The relationship between V_OC_ and light intensity can be expressed by the following formula:^[^ ^58^ ^]^ (4) $V_{\mathrm{oc}}\propto n\left(\frac{k_{B}T}{q}\right)\ln(I)$, where 𝑛, K_B_, T and q represent the ideal factor reflecting defect‐assisted recombination in PSCs, the Boltzmann constant, temperature (in Kelvin), and elementary charge, respectively. From the fitting of the curves in Figure 5e, the ideal factor n for the control PSC is 1.35, whereas that for the target PSC decreases to 1.20, which indicates that defect‐assisted recombination in the target PSC is effectively suppressed. Given that FF is influenced by factors such as charge extraction, non‐radiative recombination, and transport,^[^ ^55^ ^]^ we investigate in the following the origin of its enhancement in Co‐SAM‐based PSCs. Charge transport at the interface was further investigated using electrochemical impedance spectroscopy (EIS). We used the equivalent circuit model shown in the illustration of Figure 5f to analyze the Nyquist plot of the PSCs with the fitted parameters present in Table S6 (Supporting Information). The recombination resistance (R_rec_) and series resistance (R_s_) for the target PSC are 13087 and 11.88 Ω, respectively, compared to 7535 and 16.66 Ω for the control PSC. The target PSC displays a higher R_rec_ and a lower R_s_, indicating suppressed charge recombination at the perovskite interface and improved transport properties.^[^ ^59^ ^]^ The loss between the Shockley–Queisser‐limited FF and the measured FF arises from non‐radiative and charge transport losses. Following Note S3 (Supporting Information), we calculate the maximum FF without charge transport losses in Figure S20 (Supporting Information).^[^ ^60^ ^]^ It is clear from Figure S20 (Supporting Information) that both the non‐radiative recombination losses and charge transport losses in target PSC are lower, which are attributed to reduced recombination at the HTL/perovskite interface, consistent with the results of PL, SCLC, and EIS measurements. Additionally, Co‐SAM substrates significantly reduce leakage current derived from dark current measurements, as shown in Figure 5g. This reduction is primarily attributed to the uniform Co‐SAM coverage, which promotes high‐quality perovskite film growth and suppresses non‐radiative recombination caused by direct contact between ITO and the perovskite.^[^ ^61^ ^]^ In conclusion, Co‐SAM effectively mitigates interface defects in the PSCs with enhanced device performance.

To evaluate Co‐SAM's impact on PSC stability, we tracked normalized PCE evolution for unencapsulated PSCs stored in a nitrogen glove box, as shown in Figure 5h. Initial J–V parameters of the PCSs used in this measurement are provided in Table S7 (Supporting Information). After 3000 h, the target PSC retained 90% of the initial PCE, significantly exceeding the control PSC's less than 75%. Moreover, our initial stability measurements indicate that the Co‐SAM‐based PSCs exhibit enhanced light a thermal stability (Figure S21, Supporting Information). We attribute this enhanced stability to the dense, smooth Co‐SAM layer, which prevents direct perovskite‐substrate contact and mitigates ion migration at the buried interface, thereby improving device longevity.

## 3 Conclusion

In summary, we developed a bifacial buried interface passivation strategy for inverted PSCs by engineering a Co‐SAM on NiOx through BPPA incorporation into Me‐4PACz. The addition of BPPA suppresses Me‐4PACz aggregation, enabling uniform and high‐coverage Co‐SAM formation on NiOx. Crucially, BPPA's phosphonic acid groups coordinate with NiOx to inhibit Ni^2+^ formation while promoting Ni^3+^ species, thereby enhancing HTL conductivity and hole mobility. This simultaneously optimizes energy level alignment at the perovskite/HTL interface, reducing carrier accumulation. The Co‐SAM's reduced roughness and improved wettability further facilitate perovskite crystallization, minimizing interfacial defects and bulk film traps. Concurrently, BPPA's ‐PO_3_H_2_ groups passivate Pb^2^⁺ defects at the buried interface, suppressing non‐radiative recombination. Collectively, this synergistic interfacial engineering approach enhances charge extraction kinetics and overall device performance. Consequently, the PCE of the champion PSC increased from 24.72% to 26.35%, driven by significant gains in V_OC_ and FF. Unencapsulated Co‐SAM devices also demonstrated enhanced operational stability, retaining 90% of the initial efficiency after 3000 h in a nitrogen‐filled glovebox. This work establishes that BPPA/Me‐4PACz‐derived Co‐SAMs on NiOx synergistically optimize buried interface contacts, control perovskite crystallization, and passivate defects, collectively boosting both efficiency and operational stability.

## 4 Experimental Section

### Materials

*N*, *N*‐Dimethylformamide (DMF, 99.8%), anhydrous dimethyl sulfoxide (DMSO, 99.8%), chlorobenzene (CB, 99.8%), ethyl Acetate (EA, 99.5%), and isopropanol (IPA, 99.9%) were purchased from Sigma–Aldrich. Cesium iodide (CsI, 99.999%), methylammonium iodide (MAI, 99.9%), formamidine iodide (FAI, 99.9%), fullerene C_60_ (C_60_, 99.5%), bathocuproine (BCP, 99.0%), Ethanediamine dihydroiodide (EDADI, 99.5%) and 3‐(methylthio) propylamine hydroiodide (3MTPAI, 98.0%) were purchased from Xi'an Yuri Solar Co. Ltd. Lead iodide (PbI_2_, 99.999%) and methylamine hydrochloride (MACl, 99.9%) were purchased from Advanced Election Technology CO. Ltd. Nickel oxide (NiOx, 3–5 nm) nanoparticles was purchased from Liaoning Jiayun High technology Co., Ltd. Benzylphosphonic Acid (BPPA, 98.0%) was purchased from Shanghai Aladdin Biochemical Technology Co., Ltd. [4‐(3,6‐dimethyl‐9*H*‐carbazol‐9yl)butyl]phosphonic acid (Me‐4PACz, 99.0%) and [4‐(7H‐Dibenzo[c,g]carbazol‐7‐yl)butyl]phosphonic Acid (4PADBC, 98%) were purchased from Tokyo Chemical Industry Co., Ltd.

### Perovskite Precursor Solution

A 1.5 M perovskite precursor solution with the chemical formula Cs_0.05_FA_0.85_MA_0.1_PbI_3_ was prepared by dissolving 19.5 mg of CsI, 23.8 mg of MAI, 15.2 mg of MACl, 219.3 mg of FAI, and 726.1 mg of PbI_2_ in 1 mL of DMF:DMSO (4:1 by volume). The precursor solution was stirred overnight before being used in the experiments. All operations were carried out in a nitrogen glove box.

### Perovskite Solar Cells Fabrication

ITO glass was sequentially ultrasonicated in cleaning agent, deionized water, and anhydrous ethanol for 20 min each. The ITO glass was then blown dry with N_2_ and subjected to ozone treatment for 20 min. Nickel oxide nanoparticles (NiOx) were dissolved in deionized water at a concentration of 15 mg mL^−1^ and ultrasonicated for 10 min. The sonicated NiOx solution was filtered through a 0.22 µm water‐based filter. The prepared NiOx solution was spin‐coated onto the ITO substrate at 3000 rpm for 30 s and then annealed at 150 °C for 10 min in air. Upon completion of the annealing, the ITO/NiOx substrate was removed from the hotplate and transferred to a nitrogen glove box within 3 min. A 0.5 mg mL^−1^ solution of Me‐4PACz or a solution with a mixing ratio of BPPA and Me‐4PACz (3:5 w/w) was spin‐coated onto the NiOx substrate at 3000 rpm for 30 s and then annealed at 100 °C for 10 min to form a SAM or Co‐SAM layer. The perovskite precursor solution was then spin‐coated onto the SAM or Co‐SAM layer using a two‐step process: from 1000 rpm for 10 s to 5000 rpm for 40 s. 150 µL of chlorobenzene (CB) was dropped as an anti‐solvent 15 s before the end of spin coating. The film was then annealed at 120 °C for 10 min to obtain a black perovskite film. The 1 mg mL^−1^ solution of EDADI and 1.4 mg mL^−1^ solution of 3MTPAI were mixed and dissolved in isopropanol, then filtered through a 0.22 µm filter. The solution was spin‐coated onto the perovskite film at 4000 rpm for 30 s, followed by annealing at 100 °C for 10 min.

For the electron transport layer, 20 nm of C_60_ was thermally evaporated onto the perovskite film surface in a vacuum of 10^−5^ Pa, followed by the deposition of a 5 nm BCP hole‐blocking layer. Finally, a 130 nm Ag electrode was thermally evaporated onto the surface in high vacuum (10^−5^ Pa), with an effective area of 0.0725 cm^2^ per cell.

### Perovskite Thin Film and Device Characterization

The surface and cross‐sectional SEM images of the perovskite thin films were obtained using a Zeiss Gemini300. XRD patterns of the perovskite thin films were measured using a Bruker X‐ray diffractometer. UPS measurements were performed using a Thermo Escalab 250Xi, and XPS measurements were conducted in a self‐built high‐vacuum system. AFM measurements were carried out using an American Dimension Fastscan instrument. Steady‐state PL and time‐resolved PL spectra were measured using a Horiba Fluoromax spectrometer. The PL measurements were conducted using an excitation wavelength of 450 nm. The UV–vis absorption spectra were acquired with a Shimadzu UV2700 spectrophotometer across a wavelength range of 500 to 900 nm. GIXRD measurements of the perovskite thin films were conducted at the Shanghai Synchrotron Radiation Facility BL03Hb with a Pilatus 2 m detector, with X‐ray energy of 10 keV and an incident angle of 0.3°, unless otherwise specified. For in situ annealing tests, the unannealed films were placed in a nitrogen‐protected device and heated, while GIWAXS images were collected with an exposure time of 2 s. Additionally, microbeam GIWAXS mapping was performed at an incident angle of 3°, with a scan range of 5 × 5 mm^2^. EIS impedance spectroscopy was performed using an electrochemical workstation, Multi Autolab. Contact angle measurements were made using an SL200KS instrument. The SCLC measurements were performed in the dark within a nitrogen atmosphere glovebox. The voltage was swept from 0 to 1.0 V at a scan rate of 0.02 V s^−1^ using a Keithley 2400 source meter. The J–V characteristics of the perovskite solar cells were measured using a Keithley 2400 source meter, equipped with a Newport Class AAA solar simulator (94023 A‐U), and calibrated with an Si solar cell with an intensity of AM 1.5G 100 mW cm^−^^2^. The EQE of the devices was recorded using LST‐QE system with a monochromator and a calibrated reference photodetector, covering a spectral range from 300 to 900 nm. The EIS measurements were carried out over a frequency range of 0.1 Hz to 1 MHz under dark conditions with an applied bias of 0.9 V. The dark current was measured in the absence of illumination with a Keithley 2400 source meter by sweeping the voltage from −1.5 to 1.5 V at a scan rate of 0.02 V s^−1^.

### Statistical Analysis

Line charts, box plots, and related visualizations were generated using the educational version of Origin. Box plots of the photovoltaic parameters and histograms of the PVSK/PbI_2_ diffraction peak areas (mean and standard deviation) were generated using the educational version of Origin. The surface roughness/potential data from AFM/KPFM were statistically analyzed using Nanoscope Analysis. SEM grain size statistical analysis was performed using Nano Measurer. The 2D GIWAXS images were processed using GIWAXS‐Tools.^[^ ^62^ ^]^ The radial integration plot of 2D GIWAXS patterns was processed using Fit2D. Composite figures or schematics were prepared using Microsoft PowerPoint. All parameters shown in the figures are described either in the main text or the corresponding figure legends.

## Conflict of Interest

The authors declare no conflict of interest.

## Supporting information



Supporting Information

## Data Availability

The data that support the findings of this study are available from the corresponding author upon reasonable request.
