# Supplementary material for: Benzylphosphonic Acid‐Engineered Compact Self‐Assembled Monolayers for Bifacial Buried Interface Passivation in High‐Performance Inverted Perovskite Solar Cells
Source: Adv Sci (Weinh). 2025 Sep 25;12(45):e12117. doi: 10.1002/advs.202512117 (PMC12677647; doi:10.1002/advs.202512117)
Supplement: Supplementary file 1 — Supporting Information [file ADVS-12-e12117-s001.pdf]

Supporting Information

Benzylphosphonic Acid-Engineered Compact Self-Assembled Monolayers for Bifacial Buried Interface Passivation in High-Performance Inverted Perovskite Solar Cells

Liujiang Zhang, Meirong Fu, Xianyuan Jiang, Ziheng Zhang, Chenyue Wang, Zhenhuang Su, Bingchen He,Lin Tang, Guanhaojie Zheng, Xingyu Gao*, Jianhua He**

L. Zhang, L. Tang, J. He
The Institute for Advanced Studies, Wuhan University, Wuhan 430072, China
E-mail: hejianhua@whu.edu.cn

L. Zhang, M. Fu, Z. Zhang, C.Wang, Z. Su, B. He, G. Zheng, X. Gao
Shanghai Synchrotron Radiation Facility, Shanghai Advanced Research Institute, Chinese Academy of Sciences, Shanghai 201204, China;
E-mail: gaoxy@sari.ac.cn; zhengguanhaojie@sari.ac.cn

L. Zhang, M. Fu, Z. Zhang
Shanghai Institute of Applied Physics, Chinese Academy of Sciences, Shanghai 201800, China

X. Jiang
School of Physical Science and Technology, ShanghaiTech University, Shanghai 201210,China

L. Zhang and M. Fu contributed equally to this work.

Supplementary Note 1

The atomic ratio of P 2*p* to Ni 2*p* was subsequently calculated based on the XPS fitting results using the following formula:

$$\frac{N_P}{N_{Ni}}=\frac{A_P/S_P}{A_{Ni}/S_{Ni}}$$

Here, A_P and A_{Ni} represent the XPS peak areas of P 2*p* and Ni 2*p*, respectively, while S_P and S_{Ni} correspond to the sensitivity factors of P and Ni. According to reference values, the sensitivity factors for P and Ni are 0.412 and 3.353, respectively.

Supplementary Note 2

The hysteresis factor (HF) is calculated by:
$HF=PCE_{reverse}-PCE_{forward}/\ PCE_{reverse}$

Supplementary Note 3

Within the Shockley-Queisser (S-Q) theoretical framework, FF losses originate from two primary sources: non-radiative recombination losses and charge transport losses. Higher maximum FF (FFmax) values directly correlate with reduced non-radiative recombination in devices. FFmax can be empirically calculated using the following formula:

$$FFmax=\frac{voc-\ln{(voc+0.72)}}{voc+1}$$

Where $voc=\frac{Voc}{nKBT/q}$。 [a]

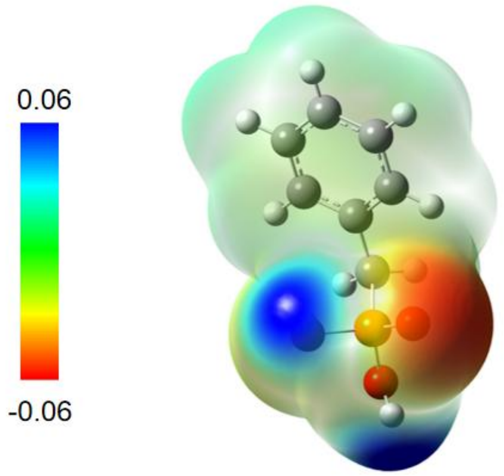

Figure S1. Electrostatic potential of BPPA molecule calculated by using Gaussian.

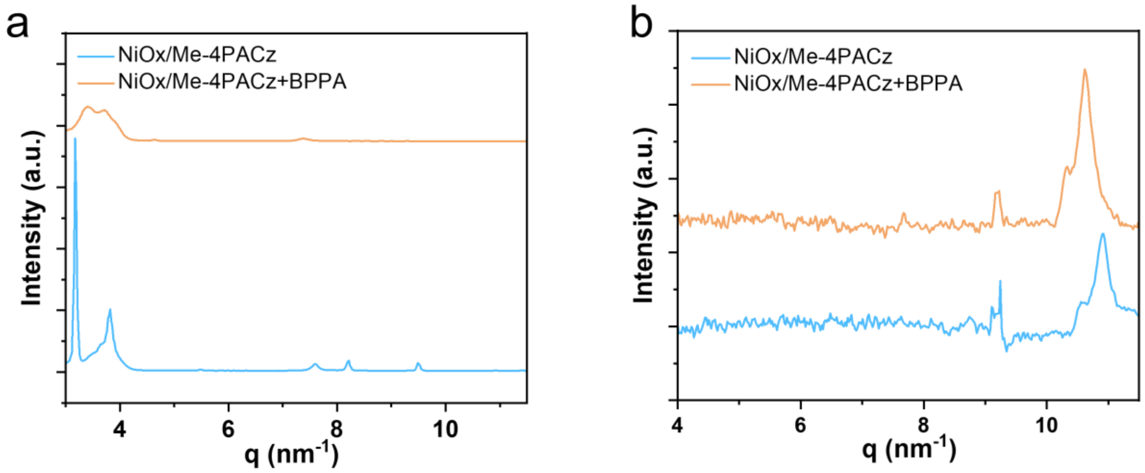

Figure S2. The 1D integrated XRD spectra derived from 2D GIWAXS patterns of SAM films with and without BPPA on NiOx along (a) out-of-plane and (b) in-plane directions.

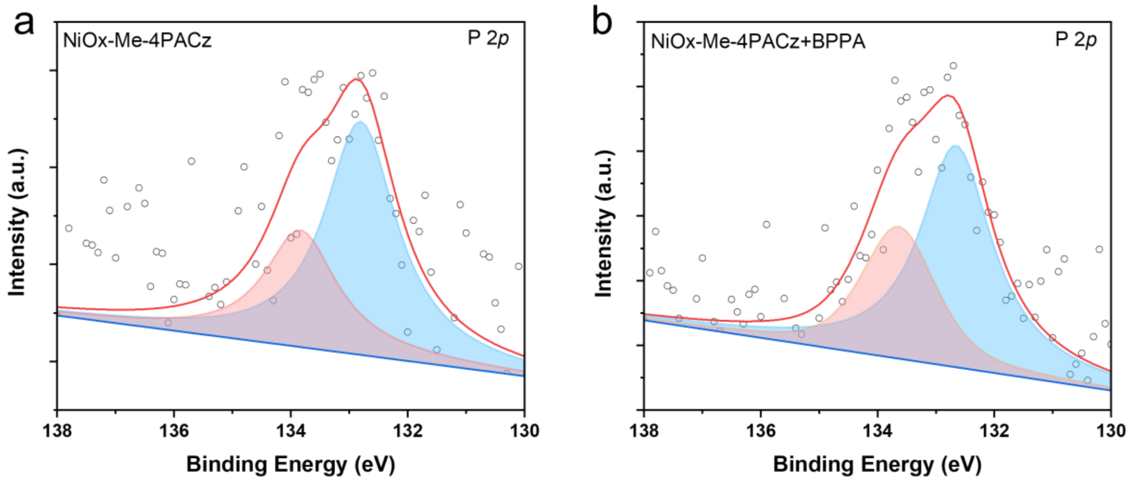

Figure S3. The P 2*p* XPS spectra of (a) NiOx/Me-4PACz and (b) NiOx/Me-4PACz+BPPA.

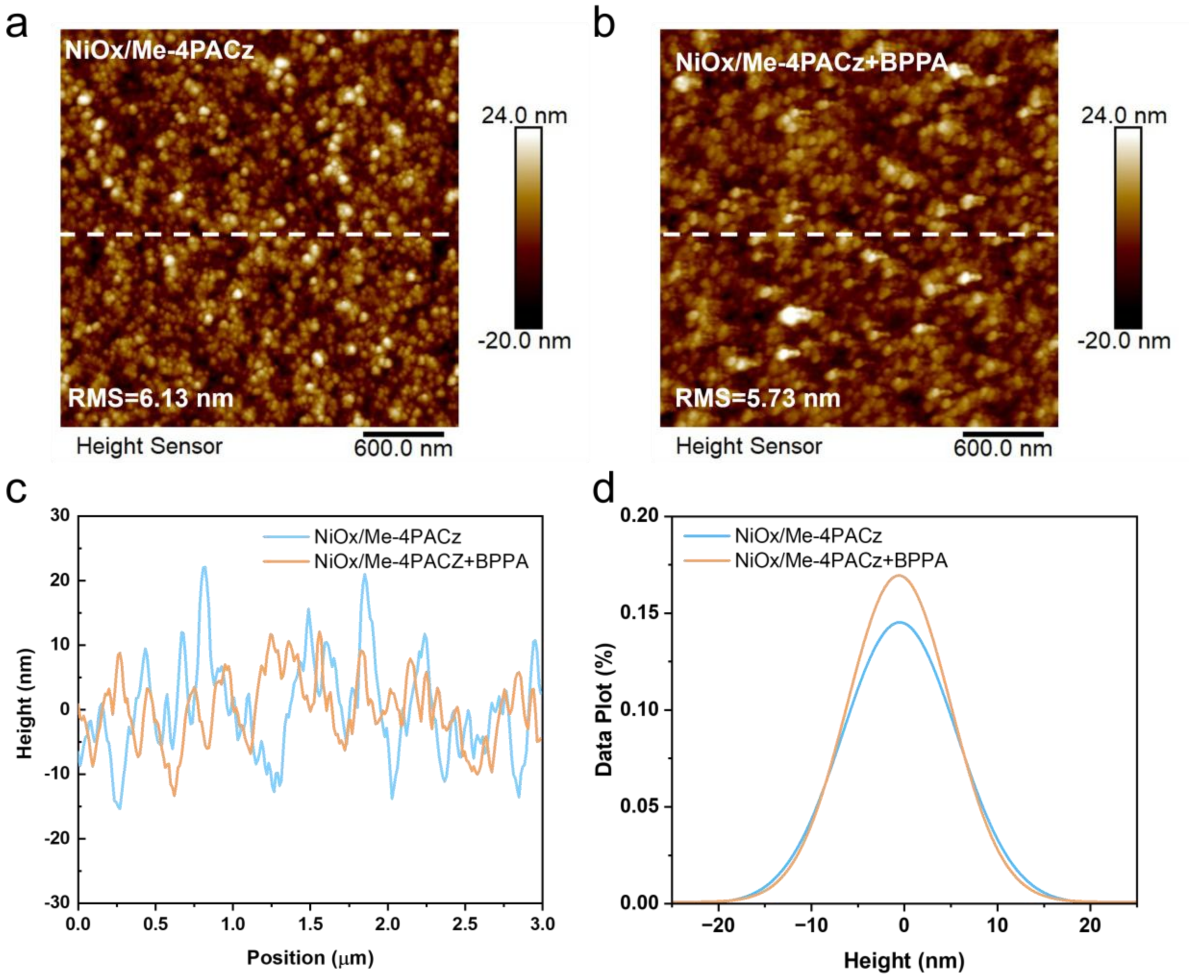

Figure S4. The AFM images of (a) NiOx/Me-4PACz and (b) NiOx/Me-4PACz+BPPA. c)The height variation along the white line in the corresponding AFM images. d) Surface roughness distribution of NiOx/Me-4PACz and NiOx/Me-4PACz+BPPA.

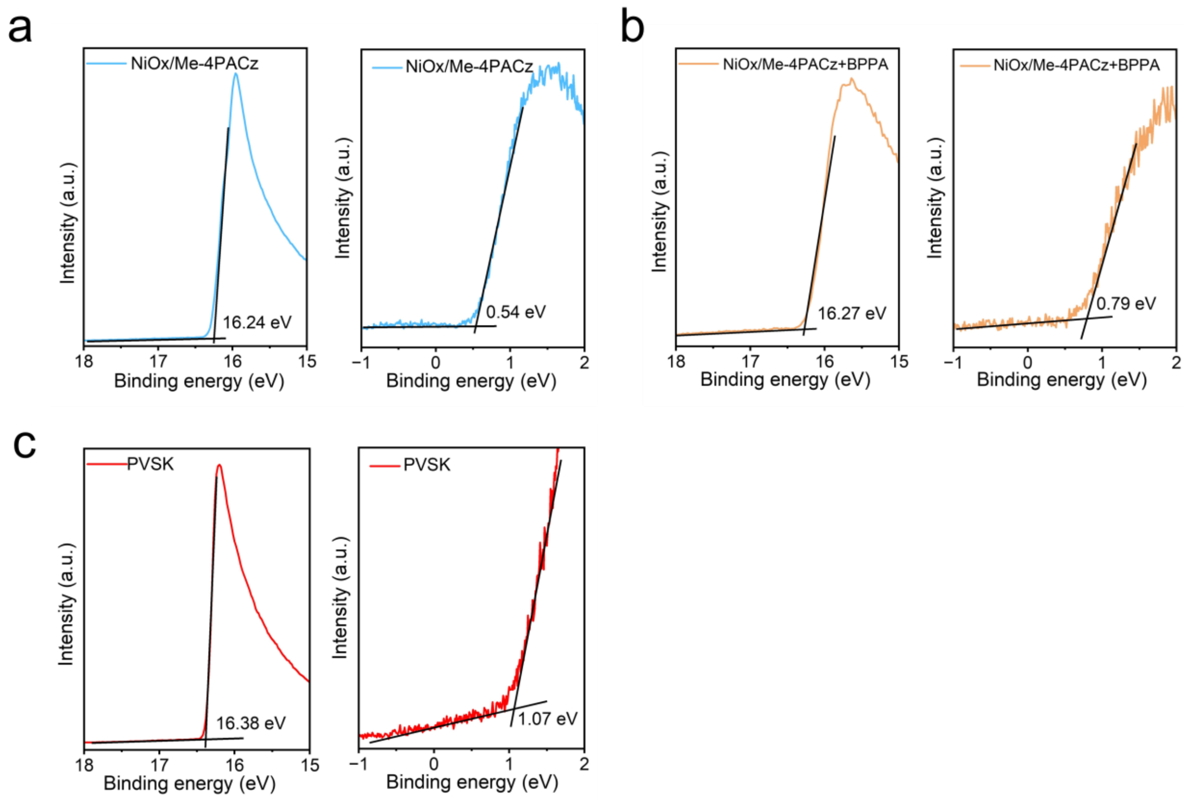

Figure S5. UPS spectra of (a) NiOx/Me-4PACz, (b) NiOx/Me-4PACz+BPPA, and (c) a perovskite film

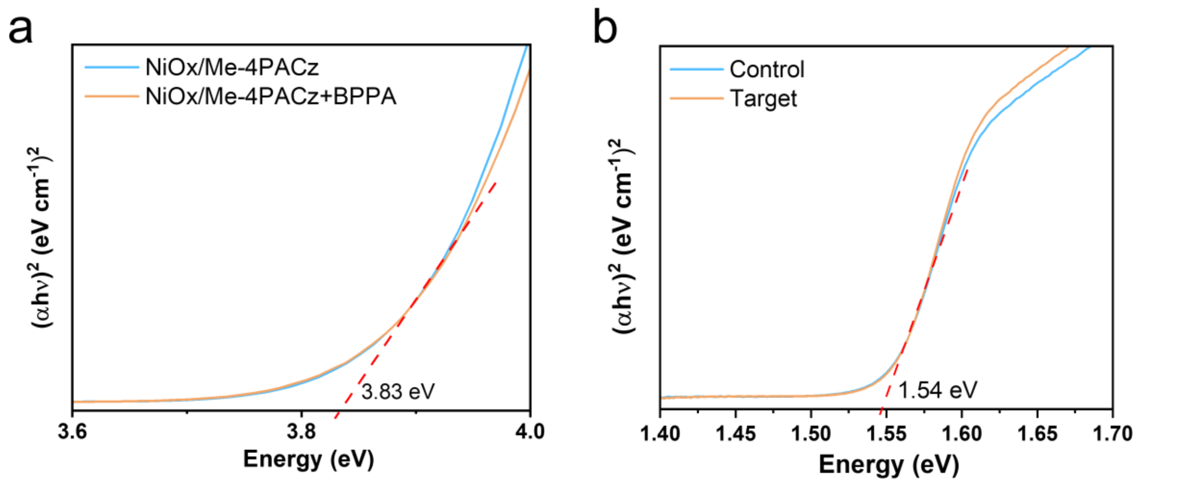

Figure S6 The Tauc plot from the UV-Vis absorption spectra of (a) HTLs and (b) perovskite films.

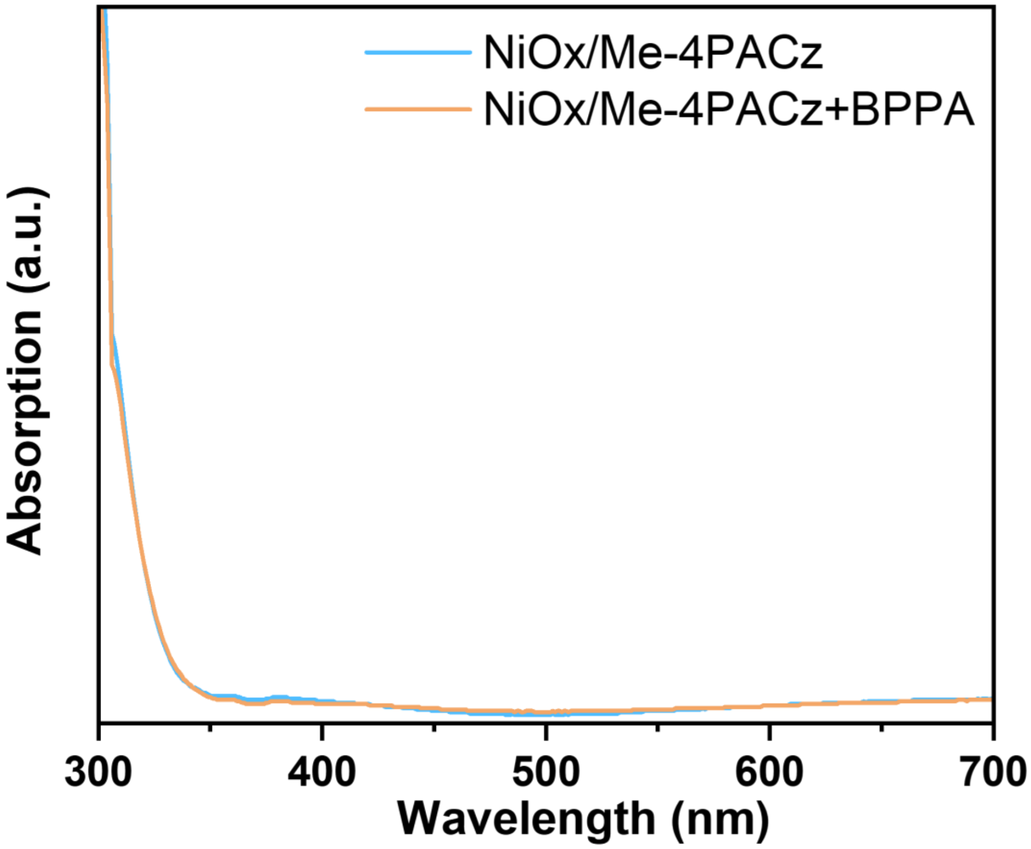

Figure S7. UV-Vis absorption spectra of NiOx/Me-4PACz and NiOx/Me-4PACz+BPPA.

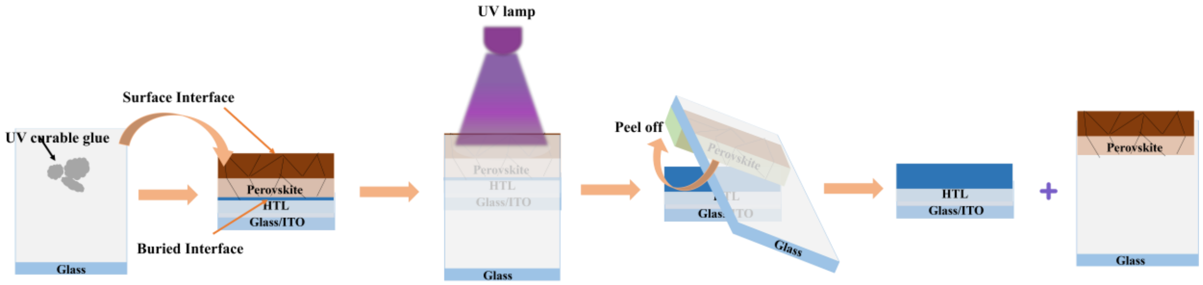

Figure S8. The schematic illustrates how the buried perovskite interface is exposed through mechanical delamination.

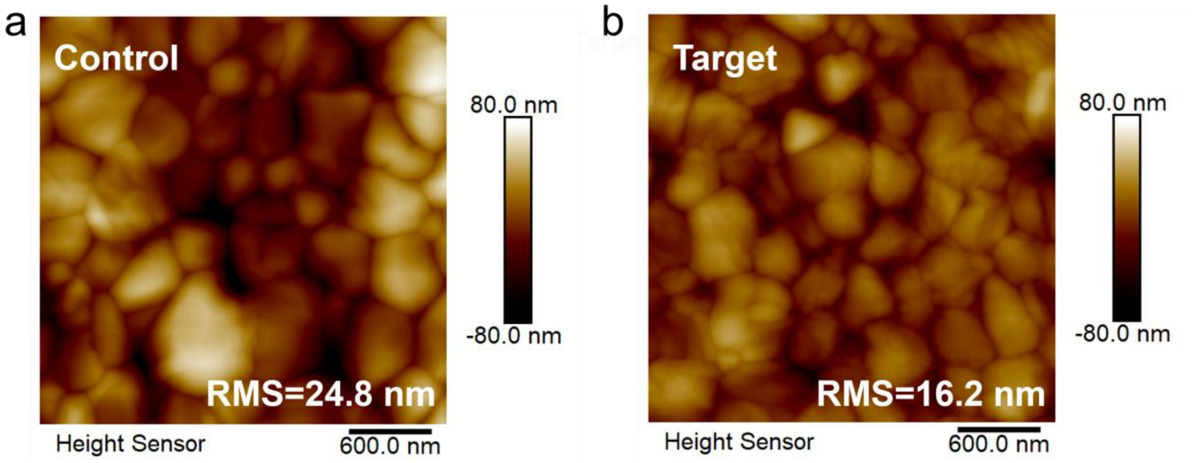

Figure S9. The AFM images of (a) a control film and (b) a target film.

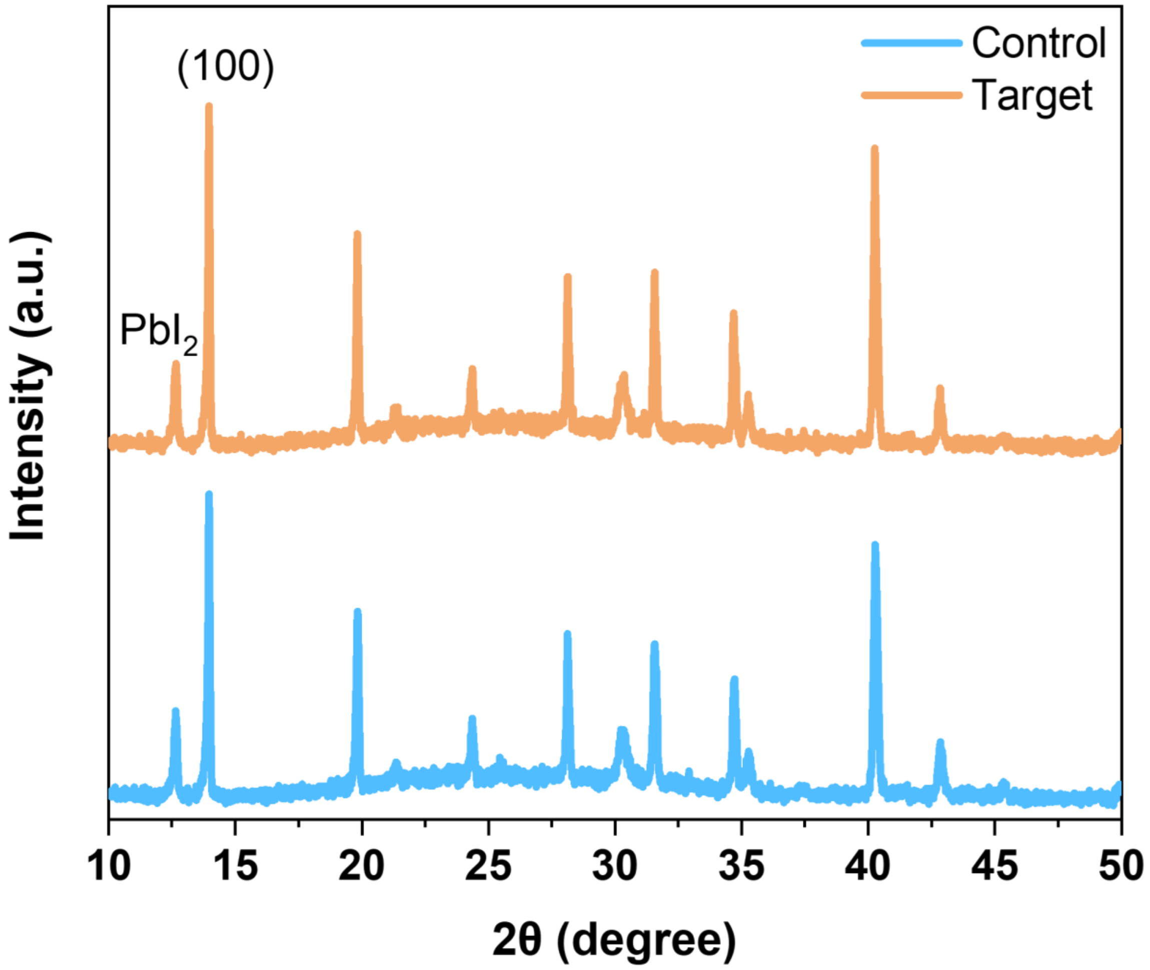

Figure S10. The XRD patterns of a control film and a target film.

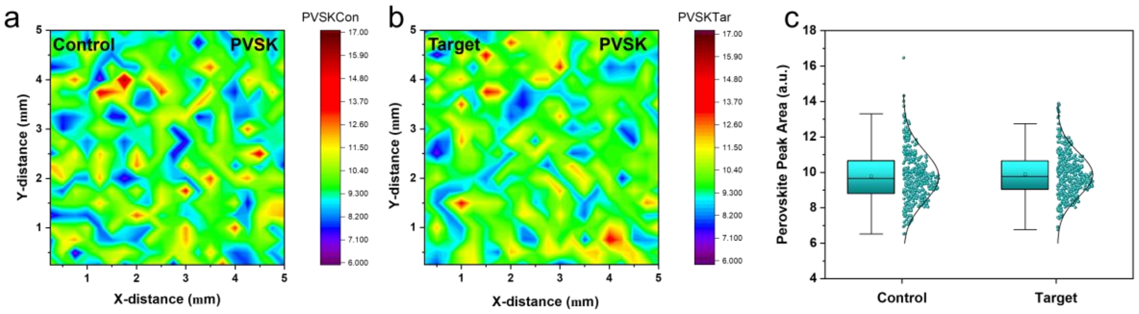

Figure S11. μ-GIWAXS mapping of perovskite (001) diffraction peak area across a 5 × 5 mm^2 area at the buried interface: control film (a) versus target film (b). The corresponding histogram (c) quantifies peak area uniformity for both films.

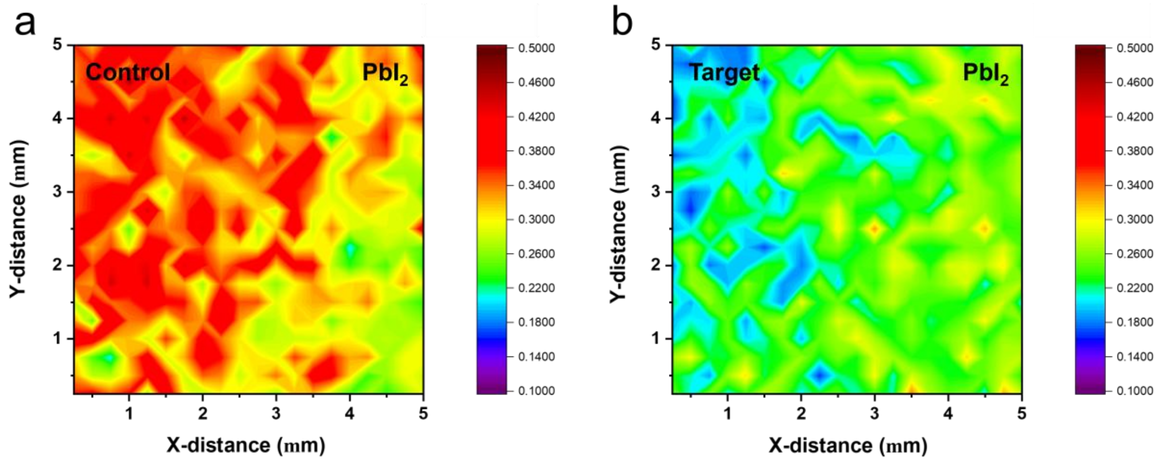

Figure S12. μ-GIWAXS mapping of PbI_2 diffraction peak area for the (a) control and (b) target films at the buried interface.

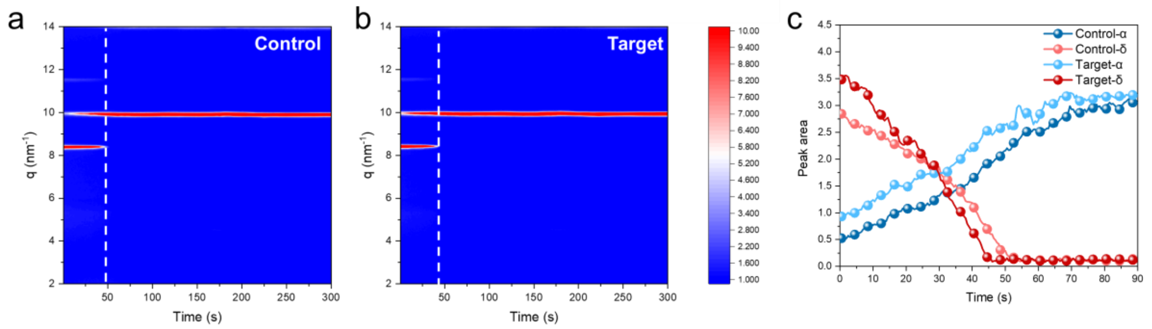

Figure S13. Contour plots of GIWAXS 1D intensity profile as functions of thermal annealing time for (a) control and (b) target perovskite films. c) The plot of the integrated intensity of the diffraction peaks of perovskite phase α and phase δ over all azimuthal angles as a function of annealing time.

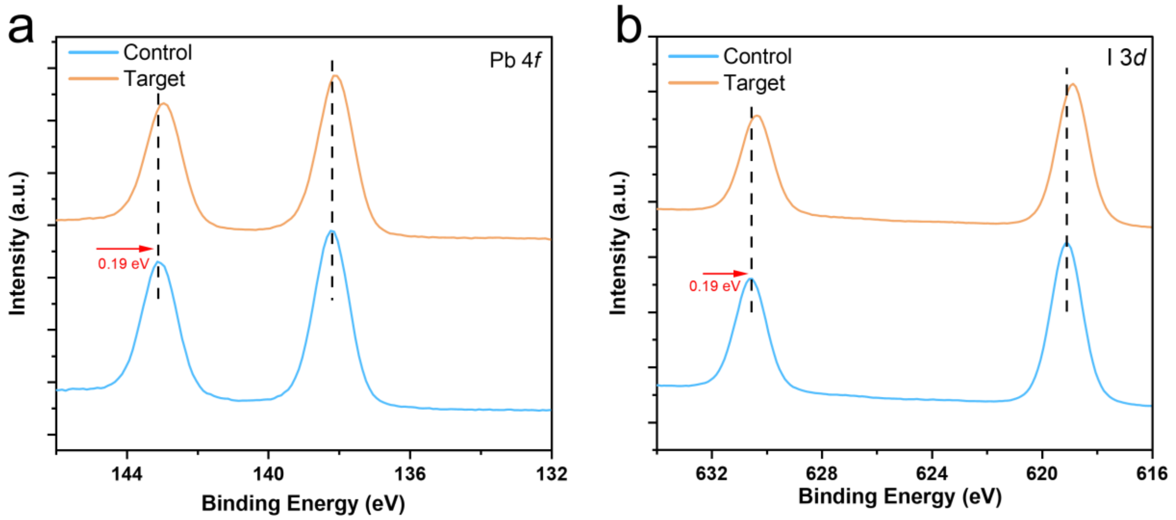

Figure S14. XPS spectra of (a) Pb 4*f* and (b) I 3*d* for control and target films, respectively.

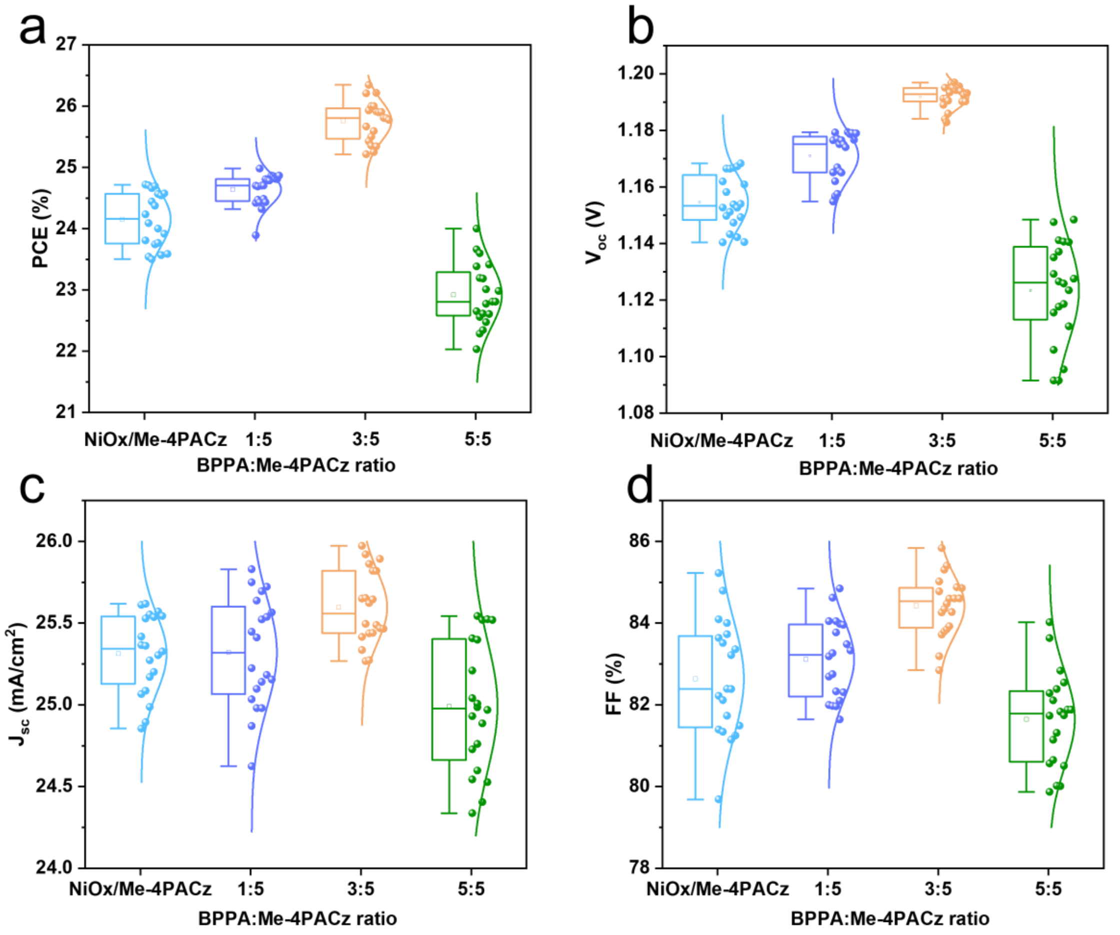

Figure S15. The statistical box charts of (a) PCE, (b) Voc, (c) Jsc and (d) FF distribution for PSCs at different BPPA mixing concentrations.

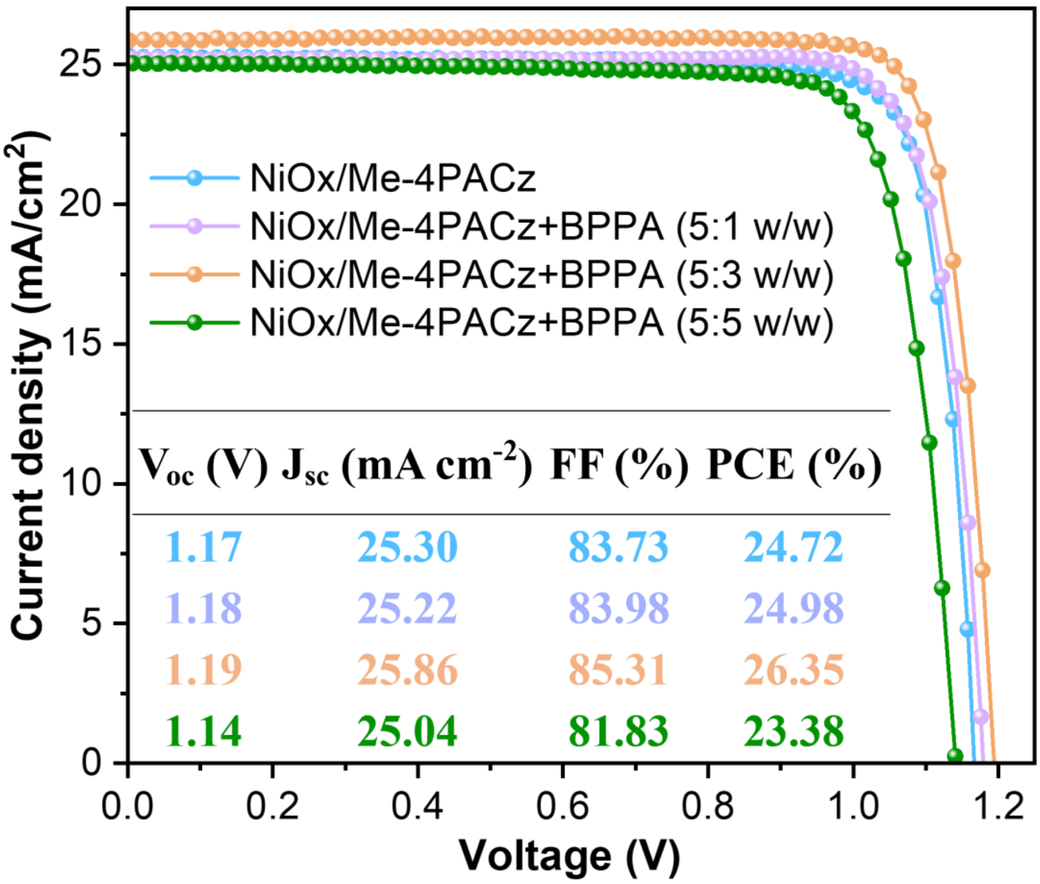

Figure S16. The optimal efficiency curves and photovoltaic parameters for the PSCs at various BPPA mixing concentrations.

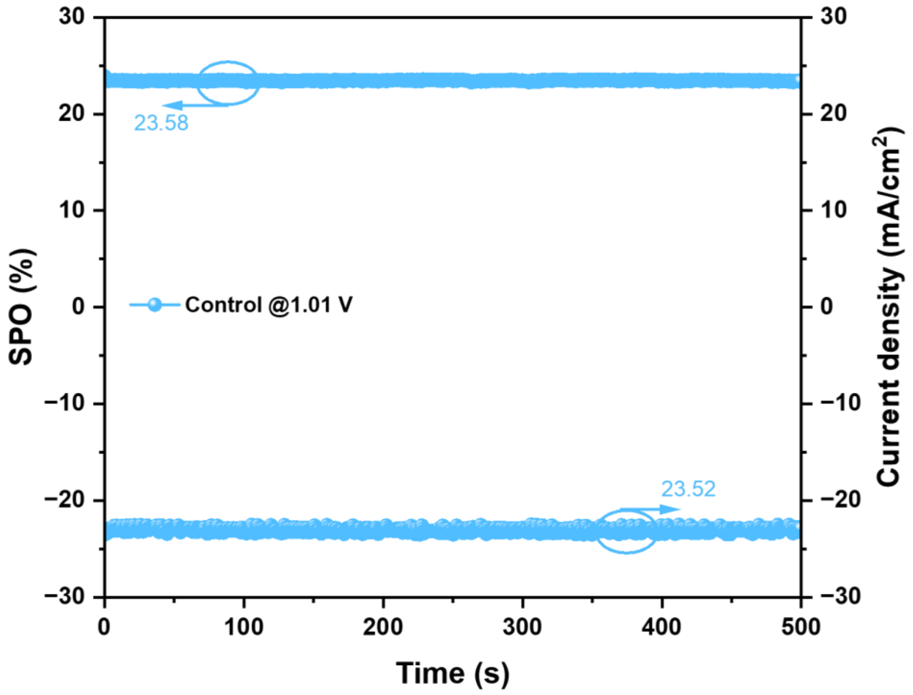

Figure S17. Steady-state output performance of PSCs based on NiOx/Me-4PACz.

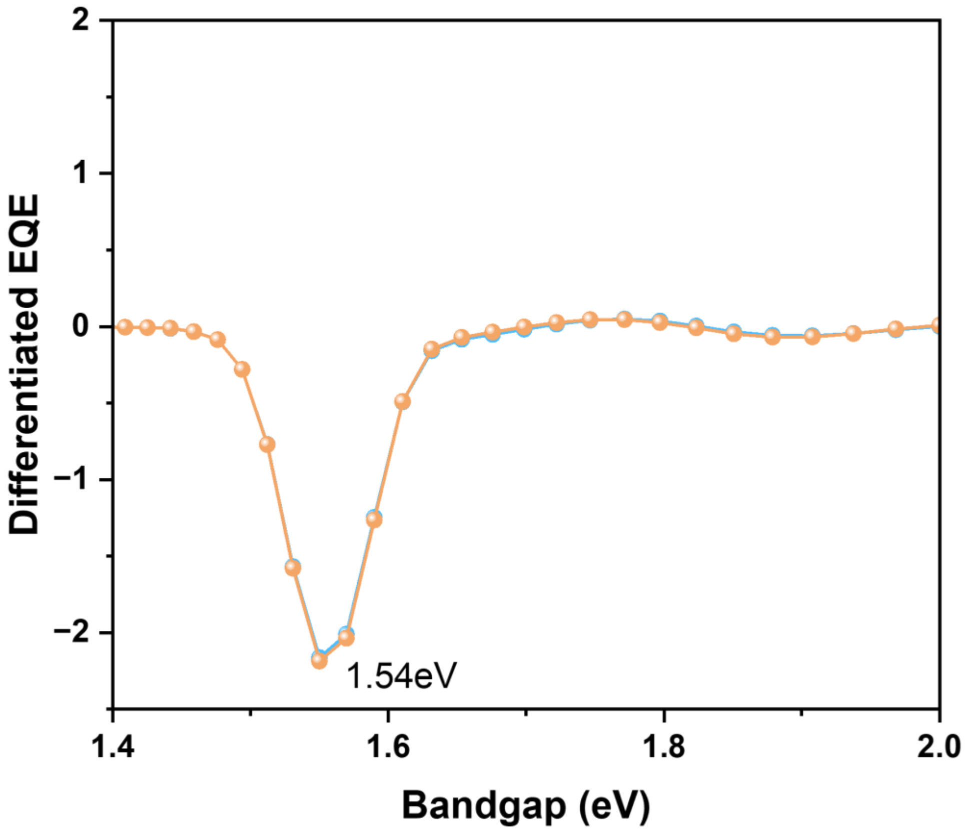

Figure S18. Eg estimation was obtained from the inflection point of the EQE spectra by identifying the maximum point (λg) of the Gaussian-like derivative $\partial EQE/\partial\lambda$.

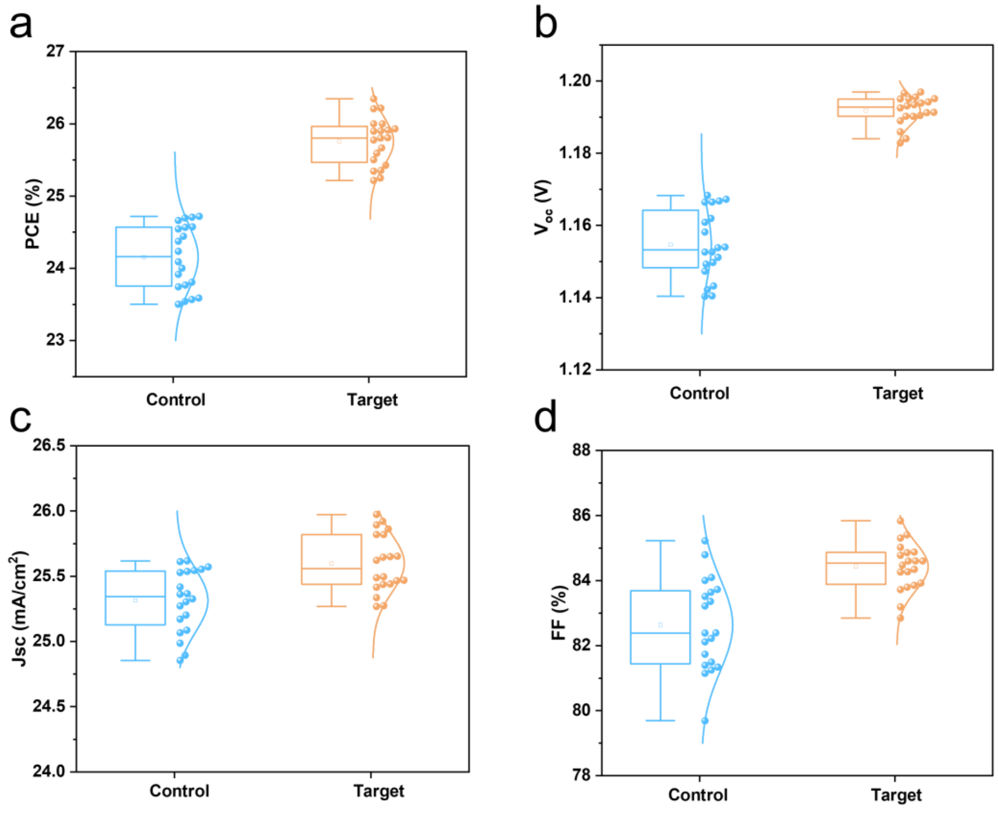

Figure S19. The statistical box charts of (a) PCE, (b) Voc, (c) Jsc, (d) FF distribution for all fabricated control and target PSCs.

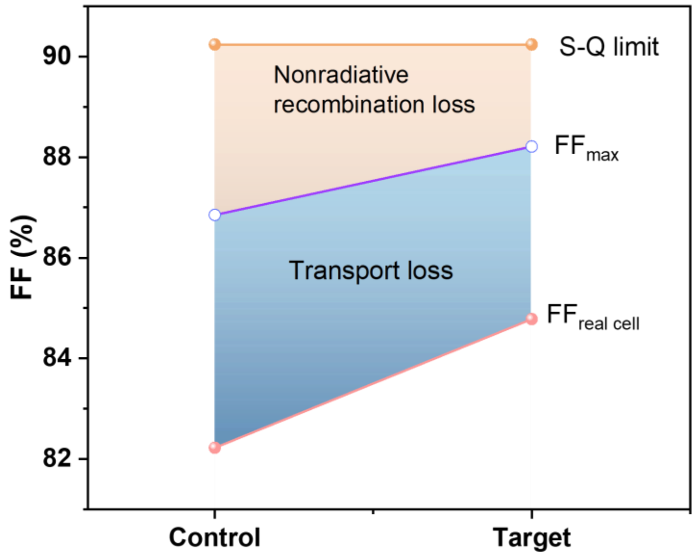

Figure S20. Detailed FF loss analysis of the inverted PSCs based on NiOx/Me-4PACz and NiOx/Me-4PACz+BPPA.

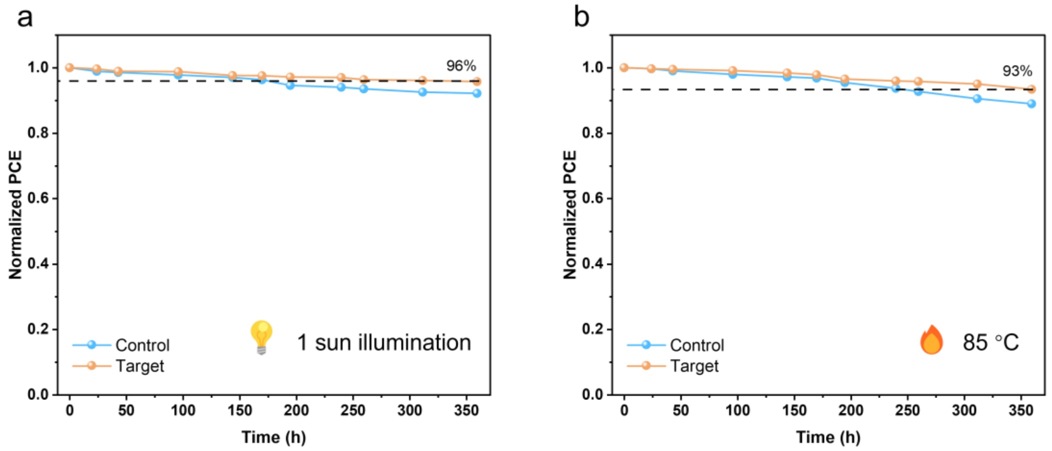

Figure S21. (a) Normalized PCE versus time of the unencapsulated PSCs under continuous light soaking in N_2. (b) Normalized PCE versus time of the unencapsulated PSCs stored in N_2 at 85°C.

Table S1. The binding energy, peak areas and relative atomic concentrations of the Ni 2*p* peaks corresponding to different oxide species in the XPS spectra.

	NiOx/Me-4PACz			NiOx/Me-4PACz+BPPA		
	**Peak position**	**Area**	**Ratio**	**Peak position**	**Area**	**Ratio**
**NiO**	853.86	872.80	44.2%	853.88	468.05	44.1%
**$Ni(OH)_2$**	855.38	612.47	30.7%	855.47	290.68	27.4%
**NiOOH***	856.62	491.93	24.6%	856.53	301.85	28.5%

Table S2. The binding energy and relative atomic concentrations of different O species in the XPS spectra.

	NiOx/Me-4PACz		NiOx/Me-4PACz+BPPA	
	**Peak position**	**Ratio**	**Peak position**	**Ratio**
**NiO**	529.29	36.2%	529.36	34.5%
**$Ni(OH)_2$**	530.54	32.0%	530.83	29.2%
**Ophosphate**	531.18	11.0%	531.46	13.4%
**NiOOH***	531.69	18.5%	532.25	20.8%
**Ochemical**	532.66		533.0	

Table S3. The binding energy and peak area of different P species in the XPS spectra.

	NiOx/Me-4PACz		NiOx/Me-4PACz+BPPA	
	**Peak position**	**Peak Area**	**Peak position**	**Peak Area**
**$2p_{3/2}$**	132.8	56.66	132.65	80.79
**$2p_{1/2}$**	133.83	28.33	133.63	40.40

Table S4. The mean and standard deviation (SD) of perovskite and PbI_2 diffraction peak areas derived from μ-GIWAXS mapping data of two films.

	PVSK (001) peak area (a.u.)		PbI_2 (001) peak area (a.u.)	
	**Mean**	**SD**	**Mean**	**SD**
**Control**	9.76	1.42	0.33	0.05
**Target**	9.87	1.23	0.24	0.03

Table S5. The fittings parameters of TRPL curves obtained from two perovskite films.

Sample	τ_1 (ns)	τ_2 (ns)	A_1	A_2	τ_{avg} (ns)
**Control**	41.51	262.26	0.59	0.32	212.39
**Target**	90.72	497.27	0.59	0.31	392.49

The average carrier lifetime (τ_{avg}) was calculated according to the equation:

$$\tau_{avg} = \frac{A_1\tau_1{}^2 + A_2\tau_2{}^2}{A_1\tau_1 + A_2\tau_2}$$

Table S6. Fitting parameters for the data from the EIS measurements.

Sample	Rs (Ω)	Rrec (Ω)
**Control**	16.66	7535
**Target**	11.88	13087

Table S7. The initial PCE of the PSCs used in the stability test (Figure 5h).

Sample	V_{oc}	Jsc (mA cm^{-2})	FF (%)	PCE (%)
**Control**	1.13	24.27	81.31	22.28
**Target**	1.14	24.79	83.87	23.71

[a] X. Li, X. Wu, B. Li, et al., Modulating the Deep-Level Defects and Charge Extraction for Efficient Perovskite Solar Cells with High Fill Factor Over 86%, *Energy & Environmental Science* **2022**, 15, 4813.
